# SARS CoV-2 mRNA vaccination exposes latent HIV to Nef-specific CD8^+^ T-cells

**DOI:** 10.1038/s41467-022-32376-z

**Published:** 2022-08-19

**Authors:** Eva M. Stevenson, Sandra Terry, Dennis Copertino, Louise Leyre, Ali Danesh, Jared Weiler, Adam R. Ward, Pragya Khadka, Evan McNeil, Kevin Bernard, Itzayana G. Miller, Grant B. Ellsworth, Carrie D. Johnston, Eli J. Finkelsztein, Paul Zumbo, Doron Betel, Friederike Dündar, Maggie C. Duncan, Hope R. Lapointe, Sarah Speckmaier, Nadia Moran-Garcia, Michelle Premazzi Papa, Samuel Nicholes, Carissa J. Stover, Rebecca M. Lynch, Marina Caskey, Christian Gaebler, Tae-Wook Chun, Alberto Bosque, Timothy J. Wilkin, Guinevere Q. Lee, Zabrina L. Brumme, R. Brad Jones

**Affiliations:** 1 Department of Medicine, Weill Cornell Medical College, New York, NY USA; 2 Immunology and Microbial Pathogenesis Program, Weill Cornell Graduate School of Medical Sciences, New York, NY USA; 3 Applied Bioinformatics Core, Weill Cornell Medical College, New York, NY USA; 4 Department of Physiology and Biophysics, Weill Cornell Medical College, New York, NY USA; 5 Faculty of Health Sciences, Simon Fraser University, Burnaby, BC Canada; 6 British Columbia Centre for Excellence in HIV/AIDS, Vancouver, BC Canada; 7 Dept of Microbiology Immunology and Tropical Medicine, The George Washington University, Washington, DC USA; 8 Laboratory of Molecular Immunology, The Rockefeller University, New York, NY USA; 9 Laboratory of Immunoregulation, National Institute of Allergy and Infectious Diseases (NIAID, NIH, Bethesda, MD USA

**Keywords:** RNA vaccines, HIV infections, Immunological surveillance

## Abstract

Efforts to cure HIV have focused on reactivating latent proviruses to enable elimination by CD8^+^ cytotoxic T-cells. Clinical studies of latency reversing agents (LRA) in antiretroviral therapy (ART)-treated individuals have shown increases in HIV transcription, but without reductions in virologic measures, or evidence that HIV-specific CD8^+^ T-cells were productively engaged. Here, we show that the SARS-CoV-2 mRNA vaccine BNT162b2 activates the RIG-I/TLR – TNF – NFκb axis, resulting in transcription of HIV proviruses with minimal perturbations of T-cell activation and host transcription. T-cells specific for the early gene-product HIV-Nef uniquely increased in frequency and acquired effector function (granzyme-B) in ART-treated individuals following SARS-CoV-2 mRNA vaccination. These parameters of CD8^+^ T-cell induction correlated with significant decreases in cell-associated HIV mRNA, suggesting killing or suppression of cells transcribing HIV. Thus, we report the observation of an intervention-induced reduction in a measure of HIV persistence, accompanied by precise immune correlates, in ART-suppressed individuals. However, we did not observe significant depletions of intact proviruses, underscoring challenges to achieving (or measuring) HIV reservoir reductions. Overall, our results support prioritizing the measurement of granzyme-B-producing Nef-specific responses in latency reversal studies and add impetus to developing HIV-targeted mRNA therapeutic vaccines that leverage built-in LRA activity.

## Abstract (web summary)

Here, the authors show in a cohort of people with HIV, COVID mRNA vaccination is followed by a transient boost in a particular profile of HIV-specific T-cell responses and a corresponding decrease in residual HIV RNA – suggesting productive immune engagement with infected cells.

## Introduction

Lifelong antiretroviral therapy (ART) has transformed HIV infection into a manageable chronic condition, but there is no safe cure^1–3^. A two-pronged strategy to reactivate latent HIV reservoirs and enhance HIV-specific cytotoxic T-lymphocytes (CTL) has been proposed to ‘shock and kill’ residual infected cells^4–6^, but trials to date have neither decreased residual HIV RNA nor reduced inducible reservoirs^7–9^. Insufficient latency reversal and/or deficiencies in CD8^+^ CTL responses are thought to be factors in these outcomes. Determining whether meaningful latency reversal has been achieved is confounded by the fact that HIV RNA (often used to measure latency reversal) does not necessarily equate to antigen expression^10^. Querying CD8^+^ T-cells for evidence of recent antigen recognition, following a therapeutic intervention, may provide a more direct way of assessing whether these cells have been engaged.

In order to optimally leverage HIV-specific CD8^+^ T-cell response measures for this purpose, we must identify which HIV antigens become visible to CD8^+^ T-cells following proviral reactivation, and which CD8^+^ T-cell functions best reveal the recognition of these antigens. Our previous in vitro and observational clinical studies support the idea that CD8^+^ T-cells specific for the early HIV gene product Nef interact with HIV reservoirs to a degree not seen in those specific for late gene products (e.g. Gag)^11,12^. We reasoned that this most likely reflects Nef-mediated immunoevasion via reduction of surface MHC-I levels^13^, where – immediately following proviral activation – a window of opportunity exists for robust MHC-I-mediated presentation of epitopes derived from early gene products, while late gene products are expressed only after this immunoevasion mechanism is in place. This has been modeled in vitro, where Nef-specific CD8^+^ T-cells can respond to reactivated cells prior to the onset of Gag expression^14^. The impact of Nef-mediated immunoevasion on reservoir surveillance in vivo has also been highlighted by a recent study associating superior Nef-mediated MHC-I downregulation in effector memory CD4^+^ T-cells with their status as a sanctuary for intact HIV proviruses on ART^15^. An additional line of evidence supporting that Nef-specific CD8^+^ T-cells can preferentially respond to residual antigen expression on ART is that these Nef-specific responses are disproportionately skewed towards granzyme-B release (gzm-B)^11^, a hallmark of recent in vivo antigen recognition as well as a key mediator of the desired cytopathic activity^16–18^. Based on the above, we hypothesized that Nef-specific CD8^+^ T-cells would both increase in frequency and be further skewed towards gzm-B production following receipt of SARS CoV-2 mRNA vaccination.

The impact of vaccination on HIV latency has been the subject of previous studies but has not yet been extended to mRNA vaccines. Günthard and colleagues demonstrated transient increases in HIV plasma viral load following influenza vaccination^19^. It was subsequently shown that the vaccine combinations of Influenza/Hep B and Pneumococcus/Hep B drove transient increases in cell-associated HIV RNA, whereas a number of other vaccines and vaccine combinations did not (e.g., measles-mumps-rubella, Varicella zoster virus, and tetanus-diptheria). Increases in cell-associated HIV RNA were accompanied by modest and transient increases in HIV-p24-specific CD8^+^ T-cell responses, as measured by IFN-γ ELISPOT, but whether this was associated with subsequent decreases in measures of HIV persistence was not assessed^20^. Here, as in other studies that reported HIV reactivation following vaccination with the recall antigens tetanus^21,22^, and cholera^23^, the relative roles of adaptive versus innate immune responses is unclear – in particular given that influenza- and tetanus-specific CD4^+^ T-cells are known to harbor portions of HIV reservoirs^24^. SARS CoV-2 vaccination provided a unique opportunity to assess the impact of a novel class of vaccines (mRNA) on the HIV reservoir, in a scenario where most study participants were antigen naïve – and to incorporate cutting-edge virologic assays, including measuring intact proviruses.

In the current study, we show that the Pfizer/BioNTech BNT162b2 SARS CoV-2 mRNA vaccine-induced HIV reactivation from the PBMCs of SARS CoV-2-naïve ART-treated people with HIV ex vivo. This was associated with innate immune sensing of mRNA and downstream activation of NFκb. This HIV reactivation occurred without detectable T-cell activation, and with minimal perturbation of host transcription. In contrast, the inactivated-virus influenza vaccine induced robust T-cell activation and host transcriptional changes, without detectable HIV reactivation in our assay. In vivo results confirmed our primary hypothesis, showing unique increases in T-cell responses targeting early gene products – predominately Nef. These increases were further pronounced when assessed by gzm-B ELISPOT. Importantly, each of the T-cell response metrics that showed significant increases following the first vaccine dose in turn showed strong correlations with subsequent decreases in cell-associated HIV RNA. Although we consider this significant decrease in a virologic measure of HIV persistence along with precise immune correlates to be an important milestone, ultimately HIV reservoir sizes were not significantly reduced. This points to additional barriers to either achieving or measuring reservoir reductions, even when a degree of success is achieved with the proximal goals of shock and kill.

## Results

### mRNA vaccines induce HIV reactivation ex vivo with minimal T-cell activation

**Fig. 1:**
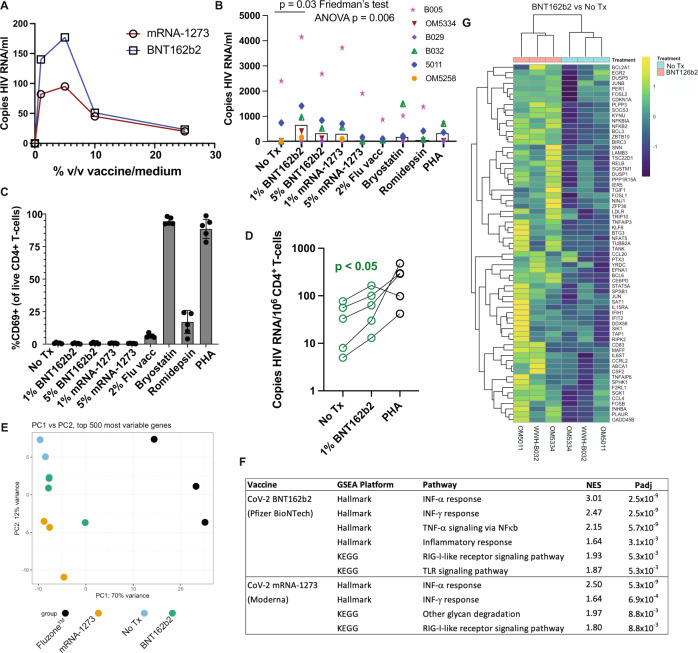
The BNT162b2 mRNA vaccine stimulates the RIG-I/TLR – TNF-α – NFκb axis and activates HIV transcription ex vivo. **A** qPCR measurements of HIV RNA in supernatants, 48 hours following ex vivo treatment of PBMCs from an ART-treated participant with the indicated concentrations of BNT126b2 (Pfizer BioNTech) or mRNA-1273 (Moderna) mRNA vaccines. **B** Extension of results from **A** to *n* = 6 ART-treated participants, adding treatments with 2% volume/volume Fluzone™ influenza vaccine, 25 nM bryostatin-1, 40 nM romidepsin, or 2 μg/ml phytohemagglutinin-L (PHA). P values were calculated by Friedman test with Dunn’s multiple comparison test (two-tailed). **C** Flow cytometry data from the same samples *n* = 5 donors harvested for (**B**) Shown are % CD69^+^ (activated) following gating on viable CD4^+^ T-cells. Data are presented as mean values +/− SD. **D** Cell-associated HIV RNA measures from the same samples as (**B**) *P* value of 0.0487 was calculated by a two-tailed paired t test between No Tx and 1% BNT162b2. **E**–**G** Bulk mRNA-seq data was generated using a subset of the samples plotted in (WWH-B032, OM5011, and OM5334). **B**, **E** Principal component analysis (PCA). The results show that transcriptional profiles of BNT126b2- and mRNA-1273-treated cells are more similar to ‘No treatment’ and to each other than Fluzone™-treated cells. **F** Gene set enrichment analyses showing pathways activated following mRNA vaccine treatments. Benjamini–Hochberg corrected P-values were calculated with the fgsea packge by a two-sided Weighted Kolmogorov–Smirnov (WGS) test. **G** Heatmap of 67 genes in the leading edge for the TNFA_SIGNALING_VIA-NFKB pathway, comparing BNT126b2 to No treatment. Source data are provided as a Source Data file.

SARS-CoV-2 mRNA vaccination induces transient systemic innate immune responses in vivo, which include the activation of TLR, RIG-I, and other inflammatory signaling pathways, providing potential latency reversal stimuli^25–27^. We therefore first assessed whether the exposure of ex vivo peripheral blood mononuclear cells (PBMCs) or purified CD4^+^ T-cells from ART-suppressed donors (Supplementary Table 1) would release HIV RNA if exposed to SARS-CoV-2 mRNA vaccines ex vivo. PBMC samples were from SARS-CoV-2 naïve individuals, with most cryopreserved prior to 2020. Cells from an initial participant were used to establish a dose-response curve, which showed peak HIV RNA release after stimulation with either the Pfizer BioNTech BNT162b2 or the Moderna mRNA-1273 vaccines at 1–5% of the culture volume – with the former showing greater induction (Fig. 1A). This approach was extended to 5 additional donors, testing the 1% and 5% doses of both mRNA vaccines, alongside 2% volume/volume of Fluzone^TM^ quadrivalent inactivated-virus influenza vaccine, as well as previously established optimal concentrations of the latency-reversing agents Bryostatin-1 and romidepsin and the mitogen phytohemagglutinin-L (PHA). We observed significant reactivation across this cohort following 1% BNT162b2 treatment (Fig. 1B**)**, with a lesser degree of overall reactivation with 5% BNT162b2 treatment (*p* < 0.05 by paired t-test) and less consistent reactivation with 1% mRNA-1273 treatment (*p* = 0.12 by paired t-test). Treatment with influenza vaccine did not induce detectable release of HIV RNA in this ex vivo system.

Established latency-reversing agents (LRAs) performed relatively poorly in this assay, which we attribute to cell-free RNA being a particularly rigorous measure of latency reversal (ex. romidepsin has previously been shown to induce detectable cell-associated but not cell-free RNA^28^). Though induction of HIV release was marginal in response to bryostatin-1 and PHA, flow cytometric analysis of CD69 expression confirmed the very high levels of T-cell activation expected with these agents^28^ (Fig. 1C). Influenza vaccine treatment was also associated with appreciable CD4^+^ T-cell activation, despite a lack of viral RNA release. Interestingly, neither of the SARS CoV-2 mRNA vaccines induced T-cell activation by this measure (Fig. 1C). SARS CoV-2 vaccines also did not appreciably impact cell viability (Supplementary Fig. 1). Using cell pellets corresponding to the supernatants assessed in Fig. 1B, we further observed that 1% BNT162b2 treatment increased levels of cell-associated HIV RNA (Fig. 1D) – corroborating LRA activity. Thus, in this ex vivo experimental system the SARS CoV-2 mRNA vaccine BNT162b2 induced HIV latency reversal without the T-cell activation typically observed with most potent latency-reversing agents.

To probe the mechanisms underlying mRNA vaccine-induced latency reversal, we purified CD4^+^ T-cells from three of the samples studied in Fig. 1B, C and subjected these to bulk mRNA sequencing (RNA-Seq) along with corresponding influenza vaccine (Fluzone^TM^) treated samples. The majority of variance between samples could be attributed to treatment effects, though with the 1% BNT162b2 samples showing relatively little divergence from untreated (No Treatment) samples (Fig. 1E). Correspondingly, treatment with the SARS-CoV-2 mRNA vaccines yielded relatively few numbers of differentially expressed genes (DEGs) compared to untreated samples: 71 DEGs for 1% BNT162b2 and 193 DEGs for Moderna’s mRNA-1273 (adj. *p* value < 0.05). The Fluzone^TM^ influenza vaccine had a substantially greater impact on the host transcriptional profile, with 3,424 DEGs.

Despite limited overall transcriptional perturbations, gene set enrichment analyses (GSEA) implicated biologically relevant signaling pathways that largely overlapped between the BNT162b2 and mRNA-1273 treatments (Fig. 1F). Retinoic acid-inducible gene I (RIG-I)-like receptors are the primary innate immune receptors of viral RNA which, when stimulated, induce type I interferons (IFN) and pro-inflammatory cytokines^29^. The results implicated both the pathway itself and the downstream IFN response in these mRNA vaccine treatments. BNT162b2 further implicated toll-like receptor (TLR) signaling. TLR-7 and TLR-8 comprise additional innate immune sensors of foreign ssRNA. They are predominately expressed by myeloid lineage cells, such as monocytes and plasmacytoid dendritic cells (pDCs), where signaling results in the release of type I IFN as well as TNF. TLR-7 agonists, including GS-9620, have been established as HIV LRAs. We have previously shown that these act predominately by driving the release of TNF, which induces NFκb activation and resulting proviral transcriptional initiation in HIV-infected CD4^+^ T-cells^30^. The observed activation of TNF signaling via the NFκb pathway implies an analogous mode of action for BNT162b2 (Fig. 1F, G). Of note, these ex vivo transcriptional perturbations are in agreement with those observed in the days following in vivo vaccination with BNT162b2 - where RIG-I like receptor signaling, TLR and inflammatory signaling, and the type I IFN response feature prominently^31^.

### mRNA vaccination drives selective increases in HIV-Nef-specific CD8^+^ T-cell responses

**Fig. 2:**
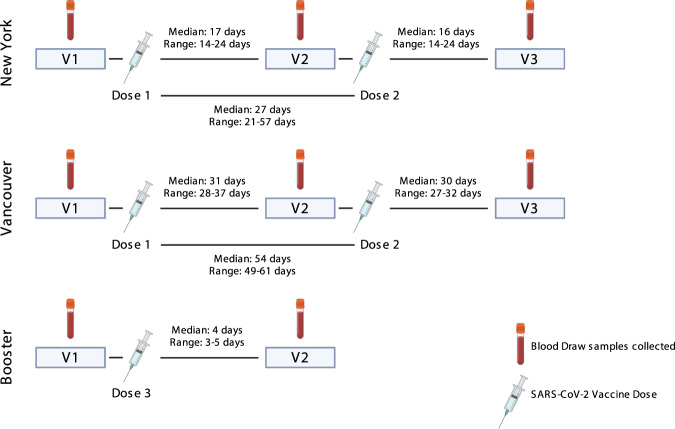
Timelines for the three clinical cohorts included in this study. Shown are blood draw dates (V1, V2, and V3) alongside timings of SARS-CoV-2 mRNA vaccinations (Dose 1, Dose 2, and Dose 3).

**Fig. 3:**
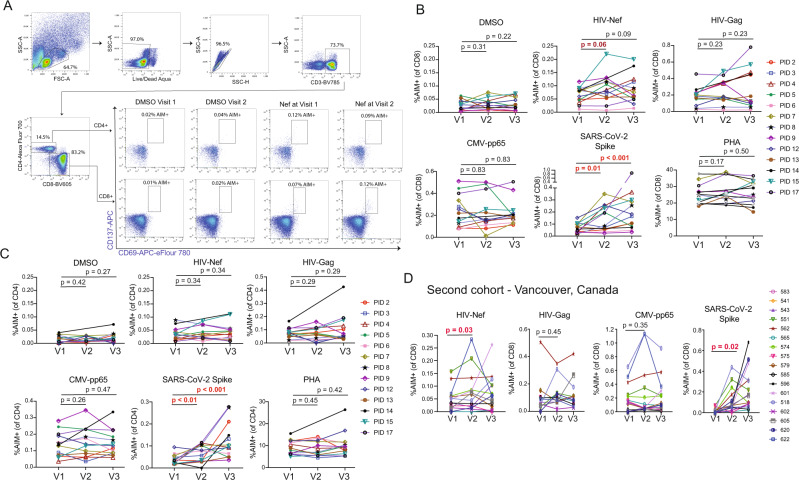
Sustained increases in SARS-CoV-2-specific CD4^+^ and CD8^+^ T-cell responses and transient increases in HIV Nef-specific CD8 T-cells following COVID mRNA vaccination. **A** Representative gating schematic for TCR-dependent activation induced marker (AIM) + populations (CD69+ CD137+) after stimulation with HIV, SARS-CoV-2 or CMV gene products. **B**, **C** Combined AIM + CD8^+^ and CD4^+^ T-cells results for *n* = 13 ARV-treated donors at baseline (V1), and ∼2 weeks after vaccine dose 1 (V2) or vaccine dose 2 (V3). CD8 + - **B**, CD4 + - **C** Data points represent means of duplicates. All responses are background (DMSO) subtracted, with the exception of the DMSO condition, which is shown as a mean of raw values. P values were calculated by one-tailed Wilcoxon matched-pairs signed rank test, adjusted for multiple comparisons using the Holm method. For the following comparisons where P values are given as <0.1 or <0.01 the exact p values are: SARS-CoV-2 Spike CD8 between V1 and V3 *P* = 0.0005, SARS-CoV-2 Spike CD4 between V1 and V2 *P* = 0.0093, between V1 and V3 *P* = 0.0007. **D** Results are analogous to panel **B**, but performed on a confirmatory cohort from Vancouver, Canada. P values were calculated by two-tailed Wilcoxon matched-pairs signed rank test, without multiple testing adjustments. Source data are provided as a Source Data file.

To assess if HIV reactivation occurred in vivo following first and second vaccine doses, we queried HIV-specific T-cell responses for evidence of antigenic stimulation using activation-induced marker (AIM) assays (assessing co-induction of CD69 and CD137) at baseline (Visit 1, V1) and ∼2 weeks after SARS-CoV-2 mRNA vaccine dose 1 (Visit 2, V2; median, range: 17, 14-24 days) and dose 2 (Visit 3, V3; median, range: 16, 14–24 days) in a cohort of 13 antiretroviral therapy (ART) treated adults with plasma HIV RNA below the limits of detection by a standard clinical assay (Table 1). Timelines for all clinical cohorts in this study are depicted in Fig. 2. As an aside, we first note that direct virologic assessments of HIV expression following initial vaccination would have been valuable – but would require sample timepoints from the days following vaccination, which were not collected. We did collect these early timepoints following mRNA vaccine boosters (third dose), where we observed significant increases in cell-associated HIV RNA (Supplementary Table 2, Supplementary Fig. 2). We did not detect cell-free HIV RNA in the plasma from these timepoints (a higher threshold for latency reversal), using the same assay as in Fig. 1A, B. These results are consistent with a moderate degree of HIV reactivation not leading to appreciable viremia. Returning to T-cell responses following initial vaccine doses, we observed the expected inductions of SARS-CoV-2-Spike-specific CD4^+^ and CD8^+^ T-cell responses following vaccine dose 1 (V2), which were further enhanced following dose 2 (V3), from means of CD8 – 0.03% AIM + (V1) to 0.08% AIM + (V2) and 0.11% AIM + (V3); and CD4 − 0.04% AIM + (V1) to 0.07% AIM + (V2) and 0.13% AIM + (Fig. 3A–C). Corresponding with this, SARS-CoV-2 anti-S serology tests showed reactivity in 2/13 individuals at V1, 10/12 at V2, and 13/13 at V3. In contrast, no significant changes were observed in HIV-Gag-specific CD8^+^ or CD4^+^ T-cells, HIV-Nef-specific CD4^+^ T-cell responses, nor cytomegalovirus (CMV)-pp65-specific responses (included as an irrelevant control) (Fig. 3B, C), nor in anti-HIV gp120 antibody levels (Supplementary Fig. 3). However, we did observe trends towards increases in HIV-Nef-specific CD8^+^ T-cell responses following first vaccine dose, from a mean of 0.06% AIM + (V1) to 0.09% AIM + (V2) – *p* = 0.06 (Fig. 3B).

**Table 1:** Study Participant Clinical and Demographic Data

Record ID	Age at Enrollment (years)	Race	Ethnicity	Sex	Gender Identity	COVID Vaccine Received	Anti-Spike Serology at Entry	Year of HIV Diagnosis	Year started multi-agent ART	Most recent CD4 (cells/ul)	Nadir CD4 if known (cells/uL)	Current ART
2	68	White	Hispanic or Latinx	Male	Man	BNT162b2	Not Detected	1993	1993	542	unknown	TAF/FTC, DTG
3	52	White	Hispanic or Latinx	Female	Woman	BNT162b2	Not Detected	2006	2006	994	unknown	TAF/FTC/BIC
4	36	White	Not Hispanic or Latinx	Male	Man	BNT162b2	Not Detected	2011	2011	1,115	unknown	ABC/3TC/DTG
5	32	Black or African American, White	Not Hispanic or Latinx	Male	Man	BNT162b2	Not Detected	2008	2008	692	10	TAF/FTC/c/EVG
6	45	White	Not Hispanic or Latinx	Male	Man	BNT162b2	Detected	2010	2010	583	6	TAF/FTC/BIC
7	27	White	Hispanic or Latinx	Male	Man	BNT162b2	Not Detected	2018	2018	591	571	TAF/FTC/BIC
8	51	Black or African American	Not Hispanic or Latinx	Male	Man	BNT162b2	Not Detected	2003	2008	154	12	TAF/FTC/c/EVG
9	61	Black or African American	Not Hispanic or Latinx	Male	Man	BNT162b2	Not Detected	1994	1996	574	unknown	TAF/FTC/c/DRV
12	29	White	Not Hispanic or Latinx	Male	Man	BNT162b2	Detected	2016	2016	760	640	TAF/FTC/c/EVG
13	46	White	Not Hispanic or Latinx	Male	Man	mRNA 1273	Not Detected	2015	2015	893	276	ABC/3TC/DTG
14	52	Asian, White	Not Hispanic or Latinx	Male	Man	BNT162b2	Not Detected	1993	1995	167	unknown	TAF/FTC, DTG
15	49	Black or African American	Not Hispanic or Latinx	Female	Woman	BNT162b2	Not Detected	2005	2007	546	unknown	TAF/FTC/c/EVG
17	62	Black or African American	Not Hispanic or Latinx	Male	Transgender Woman	BNT162b2	Not Detected	2004	2004	844	282	TAF/FTC/BIC
0518	65	NA	South Asian	Male	Man	BNT162b2	Detected	1987	1996	760	10	FTC/DTG/TAF
0520	60	NA	White or Caucasian	Male	Man	BNT162b2	Not Detected	2016	2015	1230	900	3TC/DTG
0528	28	NA	White or Caucasian	Male	Man	BNT162b2	Not Detected	2017	2020	830	540	3TC/DTG
0529	28	NA	White or Caucasian	Male	Man	BNT162b2	Not Detected	2016	2016	1100	320	3TC/ABA/DTG
0532	29	NA	Latin American	Male	Man	BNT162b2	Not Detected	2017	2017	780	480	3TC/ABA/DTG
0534	48	NA	White or Caucasian	Male	Man	BNT162b2	Not Detected	1991	2010	620	260	3TC/ABA/DTG
0537	48	NA	Arab	Male	Man	BNT162b2	Detected	2018	2018	740	420	3TC/DTG
0538	25	NA	Filipino	Male	Man	BNT162b2	Not Detected	2019	2019	460	400	TDF/FTC/DTG
0541	40	NA	Middle Eastern	Male	Man	BNT162b2	Not Detected	2017	2020	1010	1010	FTC/EGV/COB/TAF
0543	32	NA	White or Caucasian	Male	Man	BNT162b2	Not Detected	2018	2018	970	490	FTC/TAF/BCG
0551	51	NA	White or Caucasian	Male	Man	BNT162b2	Detected	2009	2010	1110	560	FTC/TAF/BCG
0562	60	NA	White or Caucasian	Female	Woman	BNT162b2	Not Detected	1995	1999	920	110	3TC/ABA/ATA
0565	36	NA	Middle Eastern	Male	Man	BNT162b2	Not Detected	2016	2020	590	420	FTC/TAF/BCG
0574	61	NA	White or Caucasian	Male	Man	BNT162b2	Not Detected	1989	2011	1160	550	TDF/FTC/MKS
0575	34	NA	Chinese	Male	Man	BNT162b2	Not Detected	2017	2015	489	330	FTC/TAF/BCG
0579	38	NA	White or Caucasian	Male	Non-binary or similar identity	BNT162b2	Not Detected	2001	2016	1800	700	3TC/ABA/DTG
0585	33	NA	Chinese	Male	Man	BNT162b2	Not Detected	2011	2011	840	10	DMP/TDF/FTC
0601	52	NA	White or Caucasian	Male	Man	BNT162b2	Not Detected	NA	2010	1560	520	3TC/NEV/ABA
0602	38	NA	Chinese	Male	Man	BNT162b2	Not Detected	2003	2005	800	170	FTC/TAF/BCG
0605	32	NA	Latin American	Male	Man	BNT162b2	Not Detected	2016	2016	440	290	3TC/ABA/DTG
0620	33	NA	Southeast Asian	Female	Woman	BNT162b2	Not Detected	2016	2020	870	750	FTC/TAF/BCG
0622	37	NA	White or Caucasian	Female	Woman	BNT162b2	Not Detected	2003	2007	320	160	TDF/FTC/DRV/COB

*ABC* abacavir, *BIC* bictegravir, *c* obicistat, *DRV* darunavir, *DTG* dolutegravir, *EVG* elvitegravir, *FTC* emtricitabine, *RPV* rilpivirine, *TAF* tenofovir alafenamide, *3TC* lamivudine.

We took two approaches to further assess this increase in Nef-specific CD8^+^ T-cell responses following the first vaccine dose, given that it was on the margin of statistical significance: i) We performed these same AIM assays on an independent cohort (*n* = 15) and ii) We re-assessed T-cell responses in this original cohort by gzm-B ELISPOT, a more selective readout of cells that have recently encountered antigen in vivo^16,32,33^. Our independent validation cohort was based in Vancouver, Canada, and had samples from baseline (pre-vaccine, V1) and ∼4 weeks after both SARS-CoV-2 mRNA vaccine dose 1 (V2; median 31, range 28–37 days) and dose 2 (V3; median 30, range 27–32) (Table 1 & Fig. 3)^34^. Due to Canada’s decision to delay second SARS-CoV-2 vaccine doses due to limited initial vaccine supply the time between first and second vaccine doses was significantly longer for the Vancouver (median 54, range 49–61 days) compared to the New York cohort (median 27, range 21–57 days). As such, the V3 results are not directly comparable. For the Vancouver cohort, we observed a significant increase in HIV-Nef-specific CD8^+^ T-cell responses following the first vaccine dose from a mean of 0.05% AIM + (V1) to 0.09% AIM + (V2) – *p* = 0.03 (Fig. 3D). No such increases were observed for HIV-Gag or CMV-pp65, while SARS-CoV-2-Spike-specific CD8^+^ T-cell responses were induced as expected (Fig. 3D). Thus, AIM results from the validation cohort further support our hypothesis by showing unique boosting of HIV-Nef-specific CD8^+^ T-cell responses following the first dose of SARS-CoV-2 vaccine.

**Fig. 4:**
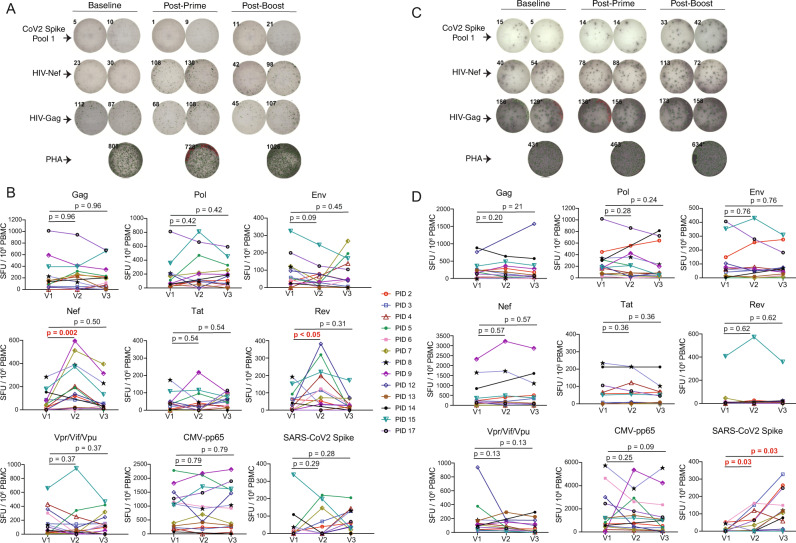
Transient increases in granzyme-B T-cell responses to early HIV-gene products following the first dose of SARS-CoV-2 mRNA vaccination. **A**, **C** Representative ELISPOT results measuring granzyme-B (Gzm-B) (**A**), or IFN-γ (**C**). Peptide stimulations are plated in duplicates. **B**, **D** Combined ELISPOT results ART-treated donors at baseline (V1), and ~2 weeks after vaccine dose 1 (V2) or vaccine dose 2 (V3). Gzm-B – **B**, IFN-γ – **D** *n* = 13 donors for and V3, and *n* = 12 for V2 (donor 14 did not provide a V2 sample). Data points represents means of duplicates, following background (DMSO) subtraction. *P* values were calculated by one-tailed Wilcoxon matched-pairs signed rank test, adjusted for multiple comparisons using the Holm method. The exact *P* value for Gzm-B Rev between V1 and V2 is 0.02. Source data are provided as a Source Data file.

Both effector and memory CD4^+^ and CD8^+^ T-cells readily produce IFN-γ in vitro in response to their cognate antigens, evidencing either past or ongoing antigen exposure. Granzyme-B (gzm-B) production following short-term in vitro stimulation, however, is a distinguishing feature of virus-specific effector CD8^+^ T-cells that have recently encountered antigen in vivo, through either infection or vaccination (with induction from memory CD8^+^ T-cells requiring >24 h of in vitro stimulation)^16–18^. To further test the hypothesis that SARS-CoV-2 mRNA vaccination can reactivate HIV expression, we assessed gzm-B and IFN-γ responses in parallel by ELISPOT, using peptide pools spanning each of: HIV-Gag, HIV-Env, HIV-Pol, HIV-Nef, HIV-Tat, HIV-Rev, HIV-Vif/Vpr/Vpu (combined pool), CMV-pp65, and SARS-CoV-2-Spike^11,35^. Amongst the HIV-specific responses, increases were uniquely observed in gzm-B-producing responses to the early gene products Nef and Rev – spiking between V1 (baseline) and V2 (vaccine dose 1) from means of 71 and 50 spot forming units (SFU)/10^6^ PBMC to 220 and 129 SFU/10^6^ PBMCs, respectively (Nef – 3.1-fold increase, *p* = 0.002, Rev – 2.6-fold increase, *p* < 0.05) (Fig. 4A, B). No inductions of Nef- or Gag-specific T-cell responses were observed following SARS-CoV-2 mRNA vaccination in a cohort of HIV-negative individuals, ruling out HIV- SARS-CoV-2-Spike cross-reactivity as a driver of these increases (Supplementary Fig. 4A, B). As expected, we observed inductions of SARS-CoV-2-Spike-specific T-cell responses as measured by either gzm-B (means: V1 − 42 SFU/10^6^ PBMCs, V2 − 51 SFU/10^6^ PBMCs, V3 − 71 SFU/10^6^ PBMCs) or IFN-γ (means: V1 – 13 SFU/10^6^ PBMCs, V2 − 47 SFU/10^6^ PBMCs, V3 − 114 SFU/10^6^ PBMCs), and a lack of significant changes in CMV-pp65-specific responses (Fig. 4). Note that the somewhat weak gzm-B character of the SARS-CoV-2 responses is likely attributable to their predominant CD4 component (Fig. 3C). Correlations between HIV-specific T-cell responses as measured by AIM or by ELISPOT are given in Supplementary Fig. 5 and show a strong positive correlation between Nef-specific T-cell responses at V2 as measured by gzm-B ELISPOT versus AIM (CD8) (Spearman’s ρ = 0.93, *p* < 0.0001). These findings support the hypothesis that the first dose of SARS-CoV-2 mRNA vaccine induces HIV reactivation that is preferentially sensed by early-gene-product specific T-cells, driving an effector functional profile.

Although only Nef, and to a lesser extent Rev, specific T-cell responses showed significant changes across the cohort by gzm-B ELISPOT, we did observe some fluctuations in other responses in individual donors. For example, PIDs 9 and 15 showed modest increases in CMV-pp65 responses between V1 and V2. This may have been attributable to bystander activation following SARS-CoV-2 mRNA vaccination or to natural dynamics in responses. Since we are not aware of any study that has monitored variation in gzm-B-specific responses over ∼2 week intervals, we assessed this by collecting samples from a cohort of PWH that did not receive a vaccine and measured responses at similar intervals to our vaccine cohorts (V1 – V2 = 21, 16-25 days and V2 – V3 = 18, 13–25 days) (Supplementary Table 3). Gzm-B-producing CMV-pp65-specific responses also exhibited considerable dynamics in this control cohort with one individual showing an increase of 1,153 SFU/10^6^ PBMCs and another showing a decrease of 742 SFU/10^6^ PBMCs between V1 and V3 (Supplementary Fig. 6). We conclude that the sporadic changes in gzm-B-producing CMV-pp65-specific in our vaccine cohort cannot be attributed to the vaccine. Future studies may ask whether gzm-B-producing CMV-pp65-specific response dynamics reflect CMV reactivation events. Levels of CD69 expression on total CD4^+^ T-cells also did not change across study visits (Supplementary Fig. 7), indicating a lack of substantial bystander activation. Together, these data confirm that – while numerous events may cause fluctuation in gzm-B-producing responses – increases in HIV early gene product responses are unique in being attributable to SARS-CoV-2 mRNA vaccination, as evidenced by consistent and significant increases across the cohort (Nef > Rev).

### HIV RNA decreases in association with post-vaccine Nef-specific T-cell responses

**Fig. 5:**
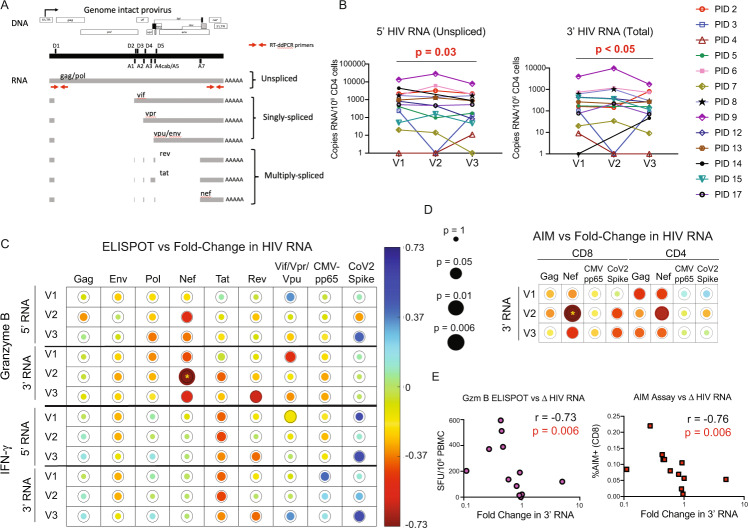
Cell-associated HIV RNA decreased across SARS-CoV-2 mRNA vaccination, in inversely correlating with Gzm-B T-cell responses to early gene products. **A** Positions of RT-qPCR primer/probes. The 3’ primer/probes target all unspliced and spliced isoforms of HIV poly(A) RNA, whereas the 5’ primer/probes only target unspliced. **B** Cell-associated HIV RNA for *n* = 13 ART-treated donors at baseline (V1), and ∼2 weeks after vaccine dose 1 (V2) or vaccine dose 2 (V3). P values were calculated by one-tailed Wilcoxon matched-pairs signed rank tests, comparing V1 to V3, without adjustments for multiple testing. **C**, **D** Depiction of Spearman correlations between proportional changes (V3/V1) in HIV RNA and magnitudes of indicated T-cell responses measured at indicated visits by ELISPOT (**C**) or AIM assay (**D**). Circle sizes are proportional to P values, and color to Spearman’s R, as indicated in the scales shown. Analyses are for the *n* = 12 individuals who completed all 3 visits (donor 14 missed V2). The rings around each circle indicate the threshold P value of 0.05. Yellow asterisks indicate correlations that were significant following correction post hoc for multiple comparisons by the false discovery rate method of Benjamini and Hochberg using the SAS MULTTEST procedure (FDR option) (see also Supplementary Tables 4 and 5). **E** Plots of the most significant correlations from **C** (left panel) and **D** (right panel). Source data are provided as a Source Data file.

The in vivo sensing of reactivated HIV by gzm-B-releasing Nef and/or Rev-specific T-cells may result in the elimination of some HIV-infected cells, specifically those that were poised for vaccine-induced reactivation. This could also explain the lack of a clear boosting effect on Nef/Rev-specific T-cell responses following second vaccine doses (Figs. 3 and 4). Alternatively, activated HIV-specific CD8^+^ T-cell responses may suppress HIV transcription, through incompletely understood mechanisms^36^. To approach the potential impact of T-cell engagement on HIV-infected cells, we first measured changes in residual cell-associated HIV RNA from baseline, following each vaccination. Levels of polyadenylated HIV RNA decreased significantly over the course of the study (V1 to V3) as measured by two different sets of primers and probes, targeting either the 5’ or 3’ region of the HIV genome. The target of the 5’ primers/probes is only present in unspliced HIV RNA, whereas that of the 3’ primers/probes is present in all splicing isoforms (Fig. 5A). 5’ HIV RNA decreased from a mean of 2027 copies/10^6^ CD4 cells at V1 to 1257 copies/10^6^ CD4 cells at V3 (1.6-fold decrease *p* = 0.03); 3’ HIV RNA decreased from a mean of 541 copies/10^6^ CD4 cells at V1 to 351 copies/10^6^ CD4 cells at V3 (1.5-fold decrease *p* < 0.05) (Fig. 5B).

Interestingly, for 3’ HIV RNA, these changes showed a strong inverse correlation with Nef-specific gzm-B-producing T-cell responses at V2 (*r* = −0.73, *p* = 0.006), and trends towards inverse correlations with Nef- or Rev-specific gzm-B-producing T-cell responses at V3 (Fig. 5C and E, Supplementary Table 4). Changes in 5’ HIV RNA showed evidence of a similar pattern, with the relationship with Nef-specific gzm-B-producing responses at V2 near the threshold of significance. This fits with the expected observations of killing of cells transcribing HIV RNA by Nef-specific gzm-B-producing T-cells given that only the 3’ primer/probes directly detect Nef-encoding spliced transcripts, whereas the 5’ primer/probes would also detect transcripts from cells where unspliced HIV RNA predominates. No correlations were observed between gzm-B-specific responses to late gene products (Gag, Pol, or Env) nor IFN-γ-producing responses to any gene product, and either HIV RNA measure (Fig. 5C, Supplementary Table 4). AIM assay results showed agreement with the gzm-B ELISPOT results, with Nef-specific CD8^+^ (*r* = −0.76, *p* = 0.006) and – to a lesser extent – CD4^+^ T-cell responses (*r* = 0.66, *p* = 0.02) correlating inversely with changes in 3’ HIV mRNA (Fig. 5D, E, Supplementary Table 5). Thus, each of the HIV-specific T-cell responses shown to be significantly increased following SARS-CoV-2 vaccine dose 1 (Figs. 3 and 4), were in turn correlated with reductions in HIV RNA. This supports a model whereby the induced T-cell responses either suppressed viral transcription^36^, and/or eliminated some of the transcriptionally competent HIV-infected cells, with the demonstrated cytotoxic functionality (gzm-B) perhaps suggesting the latter^37^. Although we have limited ability to draw kinetic inferences from our data, we propose that the associations between T-cell responses at V2 with changes in HIV RNA that manifest at V3 reflect the time it takes for these responses to expand and exert antiviral activity.

### No measurable changes in HIV reservoir size following vaccinations

**Fig. 6:**
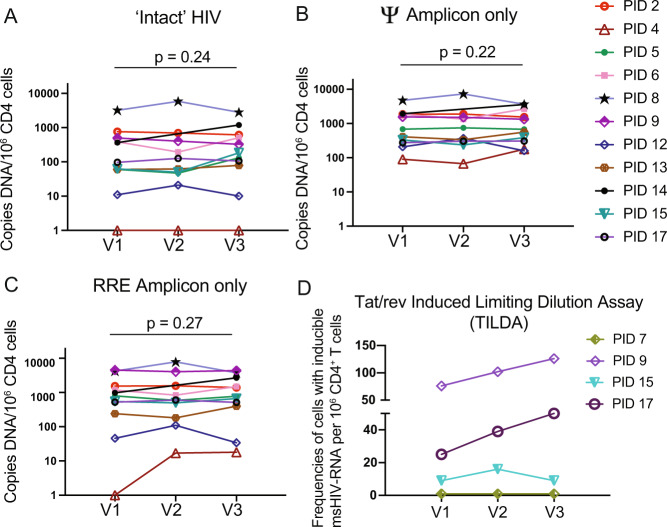
No consistent changes in HIV reservoir measures following COVID vaccinations. **A**–**C** Copies of the indicated HIV DNA species for ARV-treated donors at baseline (V1), and ~2 weeks after vaccine dose 1 (V2) or vaccine dose 2 (V3). *P* values were calculated by one-tailed Wilcoxon matched pairs signed rank test comparing V1 with V3. *n* = 13 at V1 and V3 and *n* = 12 at V2 (PID 14 missed V2). **D** Tat/rev induced limiting dilution assay results for the *n* = 4 study participants tested. Source data are provided as a Source Data file.

Measurable reductions in the frequencies of cells harboring HIV DNA would comprise more direct evidence that some infected cells had been selectively eliminated, but existing assays have important limitations. Total levels of HIV DNA provide a poor representation of the ‘HIV reservoir’ (defined as infected cells with the potential to reseed viremia), due to the fact that the large majority of integrated viral genomes are defective (e.g. large deletions)^38^. A recently developed duplex digital-droplet PCR (ddPCR) assay termed the intact proviral DNA assay (IPDA) substantially improves upon this and provides a reasonable upper estimate of genomically intact proviruses^39^. However, a further complexity is that the vast majority of these proviruses do not reactivate to produce infectious virus even after maximal in vitro stimulation^39^, and some may be limited by chromosomal context from ever reactivating^40–42^. In applying the IPDA to quantify intact proviruses, as well as the defective proviruses that yielded only packaging signal (Ψ) or rev response element (RRE) amplification, we observed a lack of significant changes in any measure across the three visits for the 11 participants that produced valid results (Fig. 6A–C) (2 individuals showed characteristic detection failures likely attributable to HIV sequence diversity in the primer or probe binding sites^43^).

We selected four individuals on the bases of clear inductions of Nef-specific gzm-B releasing responses (Fig. 4) and of sample availability, to measure the HIV reservoir using an alternative method – the Tat/rev Induced Limiting Dilution Assay (TILDA)^44^. This assay quantifies the frequencies of cells that can be induced by PMA/ionomycin to express *tat* or *rev* transcripts. Samples from two study participants (PIDs 9 and 17) showed inducible cells trending higher across visits, while this measure was unchanged in PID 15, and undetectable in PID 7. Although overall interpretation is limited by the small ‘n’ available for this assay, it is notable that PID 9 had the greatest magnitude increase in Nef-specific gzm-B responses in this study (Fig. 4), as well as one of the more marked drops in cell-associated HIV RNA in Fig. 5. Thus, these results suggest that – despite evidence for CD8^+^ T-cell engagement – reductions in cells that could be induced by PMA/ionomycin to produce transcripts were not achieved following SARS-CoV-2 mRNA vaccination.

## Discussion

Our results support a modest and transient activation of HIV expression following SARS-CoV-2 mRNA vaccination, manifesting primarily as activation of HIV-Nef-specific CD8^+^ T-cell responses. Viral RNA in blood plasma remained undetectable following vaccine boost and the ongoing presence of ARVs can be expected to block ongoing rounds of replication (further supported by our observed lack of increases in HIV DNA). Thus, there is no reason to think that this effect is clinically detrimental. Rather, the value of our observations pertains to efforts to cure HIV infection by reactivating latent reservoirs “shock” to enable immune clearance of these infected cells “kill”. Clinical trials of the shock and kill approach aimed at harnessing CD8^+^ T-cells to reduce HIV reservoirs have provided evidence for increases in viral transcription, but without reductions in measures of viral persistence nor direct evidence of antigen expression and CD8^+^ T-cell engagement^7–9^. The observations presented here advance the shock and kill concept, by providing evidence for the productive engagement of HIV-specific T-cells with their antigens in ART-suppressed donors following receipt of an mRNA vaccine – resulting in significant reductions in cell-associated HIV RNA, a measure of HIV persistence. Of key importance, we demonstrate specific immune correlates of these in vivo reductions in cell-associated HIV RNA, namely increases in T-cell responses targeting the early HIV gene product Nef - especially CD8^+^ T-cell responses and those releasing gzm-B following short-term ex vivo stimulation. These findings, in the context of an in vivo latency-reversing intervention, build upon our recent reports implicating Nef-specific T-cells as superior sensors of HIV reactivation, and gzm-B production as a hallmark of recent antigenic stimulation^16–18^. Our results indicate that incorporating these specific T-cell metrics in studies assessing latency reversal strategies will reveal evidence for in vivo engagement of T-cells that may have been missed by more conventional measures, such as Gag-specific IFN-γ responses.

A common interpretation for why past latency reversal studies have not reduced HIV reservoirs, despite increasing HIV transcription, is that CD8^+^ T-cells were not effectively engaged, either because viral RNA did not lead to antigen expression^14^, or because HIV-specific CD8^+^ T-cells were insufficiently numerous or functional^45,46^, or because these cells were unintentionally impaired by some latency reversing agents^47,48^. Certain ARVs have also been reported to impact CD8^+^ T-cell effector function^49^. The current study allows us to move beyond these factors and ask, why HIV reservoirs were not reduced despite apparent engagement of HIV-specific CD8^+^ T-cells with cytotoxic properties (gzm-B) and an associated reduction in cell-associated HIV RNA. We propose the following non-mutually exclusive possibilities. The first is that HIV-specific CD8^+^ T-cells were able to detect but not kill reservoir-harboring cells following vaccination. This is supported by mounting evidence that reservoir-harboring cells may be intrinsically resistant to killing^50,51^. Two of the genes that have been implicated in this survival and in resistance to CTL, BCL2A1 (BCL-xL)^52,53^, and BIRC3^54,55^, were upregulated by ex vivo BNT162b2 treatment (Fig. 1F) raising the possibility that resistance to killing may be enhanced alongside HIV transcription, as previously observed with some other LRAs^56^. In this model, the reduction in cell-associated HIV RNA would be attributable to CD8^+^ T-cell effector mechanisms that act to inhibit viral transcription – as has been implicated by other studies^36^. Non CD8^+^ T-cell-mediated mechanisms may also contribute to such an effect, such as vaccine-induced type I IFN responses. Another possibility is that the observed stimulation of T-cells and reduction in viral RNA was indicative of killing but involved too small of a fraction of intact or PMA/I-inducible proviruses to be detected by IPDA or TILDA. The third possibility is that appreciable CD8^+^ T-cell-mediated killing of infected cells occurred but was counterbalanced by vaccine-induced clonal expansion of HIV-infected cells. Indeed, the infected-cell landscape is complex and dynamic on ART and recent studies have implicated gradual CD8^+^ T-cell-mediated selection favoring cells with proviruses in poorly transcribed regions of the genome^42^. These possibilities can be addressed in either future mRNA vaccine studies that employ an expanded suite of reservoir quantification assays^57^, or in other settings where CD8^+^ T-cell responses show evidence of engagement. It would also be of interest for future studies to assess the impact of mRNA vaccines in individuals who exhibited pre-ART control of viremia, as their CD8^+^ T-cell responses may be preferentially able to drive reductions in reservoirs.

mRNA vaccines targeting HIV antigens are under development for both prophylactic and therapeutic settings^58^. The objectives of the latter will be to induce immune-mediated control of viral rebound upon ART interruption and/or to drive reductions in HIV reservoirs. Our results suggest that innate immune sensing of the mRNA vaccine platform itself may contribute to the latter outcome by acting as a built-in LRA, thereby providing rationale for assessing the impacts of HIV mRNA vaccines on viral reservoirs, even in lieu of combination with additional LRAs. This LRA activity is analogous to the self-adjuvant effects of mRNA that have contributed to its status as a compelling vaccine platform and - very likely - can similarly benefit from engineering to improve this activity^59^. If additional LRAs are to be combined with mRNA vaccines, their selection can also be guided to be synergistic with the LRA activity of mRNA vaccines by targeting non-overlapping mechanisms of latency. Such combinations can also be tested ex vivo as a key aspect of study design. The results of the current study also support the prioritization of Nef (and perhaps Rev) as the antigenic targets of therapeutic mRNA vaccines, as these may be more readily expressed by reactivated HIV. Although perhaps not critical, it is worth noting that the flexibility of mRNA vaccination platforms further provides a potential solution to managing Nef’s genetic variability (relative to some other viral antigens such as Gag) by allowing for the potential to tailor immunogens to those prevalent in a given geographical region, or perhaps even within a given ART-treated individual. In the context of an HIV-targeted mRNA vaccine, the ability to induce CD4^+^ T-cell responses may also contribute to impacts on the reservoir, given the importance of such responses in enhancing effector responses against HIV^60,61^. Such HIV-specific CD4^+^ T-cell responses did not show detectable boosting following SARS-CoV-2 mRNA vaccination.

In summary, our findings advance HIV cure research in two important ways. First, we have identified and provided mechanistic insights into LRA activity mediated by innate immune recognition of mRNA vaccines. Although this activity - incidental in the context of SARS-CoV-2 mRNA vaccines - was insufficient to drive measurable reductions in intact HIV DNA, there are multiple ways that future studies may intentionally optimize and leverage this built-in LRA activity to enable reservoir reductions by mRNA vaccines encoding HIV antigens. Second, we have uncovered specific measures as sensitive indicators of T-cells engagement with HIV antigens in vivo, following treatment with an LRA. Broad inclusion of these in future latency reversal studies will help identify cases where such engagement has occurred, enabling the field to evaluate and then push beyond this milestone, towards achieving and measuring reductions in HIV reservoirs.

## Methods

### Data reporting

No statistical methods were used to predetermine sample size. The experiments were not randomized and the Investigators were not blinded to allocation during experiments and outcome assessment.

### Statistics

Statistical analyses were performed in Prism Graphpad or Statistical Analysis Software (SAS). Statistical tests used are indicated in figure legends.

### Study approval and participant recruitment

Study participants with HIV were recruited at Weill Cornell Medicine’s Uptown or Chelsea Clinical Research Site. The Institutional Review Board at Weill Cornell Medicine approved this study (IRB# 21-02023358). Informed consent was obtained from all participants. Inclusion criteria were 18–89 years of age, people with HIV with sustained HIV suppression for at least one year, HIV viral load <50 copies/mL within 12 months prior to baseline visit and planned receipt of vaccination with an mRNA-based SARS-CoV-2 vaccine. Exclusion criteria included contraindication to receipt of SARS-CoV-2 vaccination, plasma HIV RNA > 200 copies/mL within one year prior to the baseline visit, known anemia with a hemoglobin <10 gm/dL, prior receipt of SARS-CoV-2 vaccination, and discontinuation of ART for 7 or more consecutive days within the prior. Blood samples used in this study were collected at the baseline visit up to 6 weeks prior to planned vaccination, 2 weeks after the first vaccine dose, and 2 weeks after the second vaccine dose. Participants completed a post-vaccine side effects survey after receipt of each dose. Results were recorded and stored in REDCap. Blood was collected via phlebotomy in gold top serum separator tubes (SST) for anti-S SARS-CoV-2 serology, pearl top plasma preparation tubes (PPT) for HIV viral load, or ethylenediaminetetraacetic acid (EDTA) tubes for PBMC processing. HIV viral load and anti-S SARS-CoV-2 serology were performed by the New York-Presbyterian Hospital clinical laboratory.

Deidentified samples from adults without HIV were obtained from the Rockefeller University (IRB protocol DRO-1006). Eligible participants were healthy adults with no history of infection with SARS-CoV-2, as determined by clinical history and confirmed through serology testing, receiving one of the two Moderna (mRNA-1273) or Pfizer-BioNTech (BNT162b2).

Our independent validation cohort consisted of a subset (*n* = 15) of study participants from a longitudinal study based in Vancouver, Canada^34^. Of the 100 PWH participants in the Vancouver cohort, 15 were selected based on sample availability and sufficient PBMC count at the time for validation purposes in this study. Ethical approval was granted through the University of British Columbia/ Providence Health Care and Simon Fraser University Research Ethics Boards. Informed consent was obtained from all participants. Vancouver participants had samples collected from baseline (pre-vaccine, V1), one month after SARS CoV-2 mRNA vaccine dose 1 (V2; median 31, range 28–37, Q1-Q3 30–32.5), and one month after vaccine dose 2 (V3; median 30, range 27–32, Q3-Q3 29–30) (Table 1 and Fig. 2). The time between doses 1 and 2 were median 54, range 49–61, Q1-Q3 51.5–57.5).

PBMC were isolated by density gradient centrifugation using Ficoll-Paque PLUS (Cytiva) and SepMate-50 tubes (StemCell Technologies). Whole blood was centrifuged at room temperature for 10 min at 400 g to separate cellular fraction and plasma. Plasma was centrifuged again at 1500 g for 10 min and then stored at −80C. Blood was diluted and centrifuged for 10 min at 1200 g. PBMC were collected and washed twice with 1X DPBS (Gibco). After the second centrifugation, cells were resuspended in 1X DPBS and counted using the Countess FL II (Invitrogen). Isolated PBMC were cryopreserved in cell recovery media containing 10% DMSO (Corning), supplemented with 90% heat-inactivated fetal bovine serum (Gibco), and stored in liquid nitrogen.

### Ex vivo latency reversal assay

Cryopreserved PBMCs were thawed and washed twice with warm R-10 medium (RPMI 1640 supplemented with 10% FBS, L-glutamine, and PenStrep, 10 mM Hepes), resuspended at 2 × 10^6^ cells/ml, in R-10 medium with 10uM T20 (NIH AIDS Reagent Program), then plated at 3 ml/well in 6-well plates. Putative latency-reversing agents (LRAs) or vaccines, and controls were added at the indicated concentrations, and cells were incubated for 24 hours at 37 °C 5% CO_2_. LRAs include 2% volume/volume Fluzone™ Quadrivalent influenza vaccine (Sanofi-Pasteur Inc., 2021–2022), 25 nM bryostatin-1 (Sigma-Aldrich, B7431), 40 nM romidepsin (Selleck Chemicals, S3020) and phytohemagglutinin-L (PHA) (Thermo Scientific/Remel, R30852801), 100 μL of resuspended cells were removed from each well and stained for flow cytometry to assess viability and activation (see below). Cells were then centrifuged at 400x *g* for 10 minutes and 2 ml media was discarded from each sample. Cells were resuspended in the remaining 1 ml media, transferred to 3 ml polystyrene tubes, and incubated for an additional 24 hours. 100 μL of resuspended cells were removed from each well and stained for flow cytometry. Cultures were then centrifuged at 400x *g* for 10 minutes and supernatants were transferred to a 1.5 ml tube. Cell fractions: CD4^+^ T-cells were enriched from PBMCs using the EasySep Human CD4^+^ T Cell Isolation Kit (Cat. No. 19052). DNA and RNA were co-extracted using the AllPrep Mini (Cat. No. 80204) from the same CD4^+^ cell sample. RNA was used for RNA-sequencing (see below). Supernatant fractions: The tubes containing supernatants were spun at 1000 × *g* for an additional 10 minutes and supernatants were transferred to clean 1.5 ml tubes then frozen at −80 °C. *Flow cytometry*: At each timepoint sampled, cells were stained with 1/100 dilutions of the following antibodies in PBS with 2% FBS, 2 mM EDTA, fixable viability dye (aqua; Thermo Fisher, cat # L34966), anti-human CD3 (clone SK7; BioLegend, cat # 344842), anti-human CD4 (clone A161 A1; BioLegend, cat # 357418), anti-human CD8 (clone RPA-T8; BioLegend, cat # 301040), anti-human CD69 (clone FN50; Invitrogen, cat # 47-0699-42), anti-human CD25 (clone BC96, BioLegend, cat # 302614). BD Cytofix/Cytoperm (BD Biosciences; 554722) was used to fix and permeabilize cells, and 1x Perm Wash buffer was used to dilute anti-HIV-1 core antigen p24 (clone KC57; Beckman Coulter, cat # 6604667, 1:100). *Viral RNA quantification:* Method 1 – Chun Lab, Fig. 1A – Viral RNA was quantified using Cobas Ampliprep/Cobas Taqman HIV-1 Test, version 2.0 (Roche Diagnostics), in quadruplicate. Method 2 – Jones Lab, Fig. 1B - Viral RNA was extracted from plasma using the QIAamp Viral RNA Mini Kit (Qiagen) according to the manufacturer’s recommendations and eluted in 60 μl nuclease-free water. Copies of HIV RNA was measured in 500 ml of cell culture supernatant using the previously described integrase single-copy assay protocol^62^. Briefly, RNA was extracted and eluted in 80 ml of water by QIAamp Viral RNA Mini Kit (Qiagen, USA). 12.5 ml of 2x buffer and 1 ml of N2 polymerase from AgPath-ID™ One-Step RT-PCR Reagents kit (ThermoFisher, USA) were mixed with 8.5 ml of extracted RNA or RNA standards, 1 ml of 10 mM forward and reverse primers, and 1 ml of 6.25 mM probe, as a total volume of 25 ml per well. Samples were analyzed on an ABI Viia7 Real-Time PCR System using the following cycling parameters: 50 °C for 10 min, 95 °C for 10 min, followed by 40 cycles of 95 °C for 15 s and 60 °C for 1 min. Cycle threshold values were compared with a validated HIV RNA standard run on each plate to determine HIV RNA concentration. The following primers and probe were used in this assay:

Forward primer: 5’-TTTGGAAAGGACCAGCAAA-3’

Reverse primer: 5’-CCTGCCATCTGTTTTCCA-3’

Probe: 5’−6FAM-AAAGGTGAAGGGGCAGTAGTAATACA-TAMRA-3’.

### RNA-sequencing

RNA was extracted using the AllPrep DNA/RNA Mini kit (Qiagen; 80204). 2-mercaptoethanol, (Bio-Rad; 1610710) was used as directed in the lysis buffer. RQ1 DNase (Promega, M6101) treatment was performed according to the manufacturer. RNA integrity was assessed by Agilent Bioanalyzer 2100, using a Total Eukaryote RNA Pico (v2.6) kit. SMART-Seq v4 Ultra Low Input RNA plus Nextera XT DNA Sample Preparation was performed. The DNA library, QC and sequencing were all performed by the Genomics Core Facility at Weill Cornell Medicine. Illumina NovaSeq 6000 was used for sequencing, using an S2 flow cell and PE 2 × 50 cycles.

### RNA-sequencing analysis

Raw reads were quality checked with FastQC v0.11.7 (http://www.bioinformatics.babraham.ac.uk/projects/fastqc/), and adapters were trimmed using Trim Galore v0.6.7 (http://www.bioinformatics.babraham.ac.uk/projects/trim_galore/). Reads were aligned to the human reference genome (GRCh38.p12) using STAR v2.6.0c^63^ with default parameters. Gene abundances were calculated with featureCounts v1.6.2^64^ using composite gene models from Gencode release 28^65^. Principle component analysis was performed using the plotPCA function from DESeq2 v1.32.0^66^, after removing study participant-specific effects with limma v3.48.0 removeBatchEffect^67^. Differentially expressed genes were determined with DESeq2 v1.32.0 using Wald tests (q < 0.05) with a two-factor model incorporating study participant. Gene set enrichment analysis was performed using fgsea v1.18.0^68^; genes were ordered between treated and untreated cells by the DESeq2 Wald statistic. Gene sets were retrieved from the Broad Institute’s MSigDB collections^69,70^. Only pathways with an adjusted *P* value < 0.05 were considered enriched. The expression heatmap of the leading-edge genes for the MSigDB Hallmark TNFA Signaling via NFKB pathway was generated using variance-stabilized data after removing study participant-specific effects, with the values centered and scaled by row.

### Activation-induced marker (AIM) assay

10 × 10^6^ PBMCs from each study participant collected at visits one, two, and three were thawed and rested for 3 h at 37 °C 5% CO2 in R-10 medium (RPMI 1640 supplemented with 10% FBS, L-glutamine, and PenStrep). PBMCs from each timepoint and participant were divided into seven conditions in duplicate at a concentration of 700,000–1,000,000 cells/condition and stimulated for 24 hours with the following whole gene product peptide pools from the NIH HIV Reagent Program at 1ug/mL: HIV-Gag (cat # ARP-12425), and HIV-Nef (cat # ARP-12545); and CMVpp65 peptide pool (cat # PM-PP65-2), PepMix™ SARS-CoV-2 Spike Glycoprotein (cat # PM-WCPV-S-1) from JPT. Phytohemagglutinin was added at 2ug/mL as a positive control, and 0.5% DMSO in PBS and R-10 media was used as a negative control. 1/200 of anti-CD107a PE ((LAMP-1) Antibody Clone H4A3 Biolegend cat# 328608) from biolegend was added to each well. Post-stimulation, PBMCs were washed in 2% FBS 2 mM EDTA- PBS and surface stained with the following fluorochrome-conjugated antibodies from Biolegend: anti-CD3-Brilliant Violet 785 clone SK7 (cat# 344842), anti-CD8-BV605 clone RPA-T8 (cat# 301040), anti-CD137-APC (4-1BB) clone 4B4-1 (cat # 309810), anti-OX40-Brilliant Violet 711 clone Ber-ACT35 (ACT35) (cat # 350030), anti-CXCR5-AF488 clone J252D4 (cat # 356912), and anti-CD4-AF700 clone A161A1 (cat # 357418); and the following from Invitrogen: Anti-CD69-APC-eFluor 780 clone FN50 (cat# 47-0699-42), as well as fixable aqua viability dye (cat # L34966). All antibodies were added at a concentration of 1/100. Cells were fixed using 4% paraformaldehyde and then analyzed on an Attune NxT flow cytometer. Data were analyzed using Flowjo software, TreeStar.

### IFN-γ and granzyme-B ELISPOT assays

Mabtech Interferon-γ (cat # 3420-2 A) and Granzyme-B (cat # 3486-2 A) enzyme-linked immune absorbent spot (ELISPOT) assays against HIV-Gag (cat # ARP-12425), HIV-Env (cat # ARP-12540), HIV-Pol (cat # ARP-12438), HIV-Nef (cat # ARP-12545), HIV-Tat (cat # ARP-12706), HIV-Rev (cat # ARP-6445), and HIV-Vpr/Vpu/Vif peptide pool (cat #s ARP-6447, ARP-6444, ARP-6446) all from the NIH HIV Reagent Program; and CMVpp65 peptide pool (cat # PM-PP65-2), PepMix™ SARS-CoV-2 VME1 (cat # PM-WCPV-VME), and PepMix™ SARS-CoV-2 Spike Glycoprotein (cat # PM-WCPV-S-1) from JPT gene product peptide pools were performed in duplicate. Multiscreen IP 96-well plates (Millipore) were coated with 0.5 ug/mL of anti-IFN-γ antibody clone 1-D1K or 7.5ug/mL of anti-Granzyme-B antibody clone MT28 in phosphate-buffered saline and incubated overnight. Plates were washed, PBMCs were added at 100,000-200,000 cells/well and stimulated with peptide pools and 0.5% DMSO and phytohemagglutinin at 2ug/mL as negative and positive controls, respectively. Plates were incubated overnight, washed and biotinylated antibody was added (anti-IFN-γ antibody clone 7-B6-1 and anti-Granzyme-B antibody clone MT8610 from Mabtech and incubated for 1 hour for IFN-γ and 2 hours for Granzyme-B plates. Plates were developed with Streptavidin-ALP from Mabtech and with Color Development Buffer (Bio-Rad, Hercules, CA). Plates were washed, dried overnight and spots were counted. Responses against whole gene product peptide pools were background subtracted (thus, nonzero responses were more than 1 time background), but no other ad hoc empirical cutoff was applied — consistent with other studies examining correlations with objectively reported T-cell responses as assessed by ELISPOT assay^11,35^.

### Duplex digital droplet PCR (intact proviral DNA assay)

Genomic DNA was isolated from CD4^+^ T-cells using the AllPrep DNA/RNA Mini Kit (Qiagen) with precautions to minimize DNA shearing. Intact HIV copies/million CD4^+^ T-cells were determined by droplet digital PCR (ddPCR) using the Intact Proviral DNA Assay (IPDA), where HIV and human RPP30 reactions were conducted independently in parallel and copies were normalized to the quantity of input DNA. In each ddPCR reaction, a median 7.5 ng (IQR 7– 7.5 ng) (RPP30) or a median 347 ng (IQR 274– 484 ng) (HIV) of genomic DNA was combined with ddPCR Supermix for Probes (no dUTPs, BioRad), primers (final concentration 900 nM, Integrated DNA Technologies), probe(s) (final concentration 250 nM, ThermoFisher Scientific) and nuclease free water. Primer and probe sequences (5′–>3′) were: RPP30 Forward Primer- GATTTGGACCTGCGAGCG, RPP30 Probe- VIC-CTGACCTGAAGGCTCT- MGBNFQ, RPP30 Reverse Primer- GCGGCTGTCTCCACAAGT; RPP30-Shear Forward Primer CCATTTGCTGCTCCTTGGG, RPP30-Shear Probe- FAM- AAGGAGCAAGGTTCTATTGTAG- MGBNFQ, RPP30-Shear Reverse Primer- CATGCAAAGGAGGAAGCCG; HIV Ψ Forward Primer- CAGGACTCGGCTTGCTGAAG, HIV Ψ Probe- FAM- TTTTGGCGTACTCACCAGT- MGBNFQ, HIV Ψ Reverse Primer- GCACCCATCTCTCTCCTTCTAGC; HIV env Forward Primer- AGTGGTGCAGAGAGAAAAAAGAGC, HIV env Probe- VIC-CCTTGGGTTCTTGGGA- MGBNFQ, anti-Hypermutant env Probe CCTTAGGTTCTTAGGAGC- MGBNFQ, HIV env Reverse Primer GTCTGGCCTGTACCGTCAGC. Droplets were prepared using the QX200 Droplet Generator (BioRad) and cycled at 95 °C for 10 min; 45 cycles of (94 °C for 30 sec, 59 °C for 1 min) and 98 °C for 10 min. Droplets were analyzed on a QX200 Droplet Reader (BioRad) using QuantaSoft software (BioRad, version 1.7.4). Four technical replicates were performed for each participant sample. Intact HIV copies (Ψ and env-RRE double-positive droplets) were corrected for DNA shearing based on the frequency of RPP30 and RPP30-Shear double-positive droplets.

### Cell-associated HIV RNA

PBMCs collected from study participants were enriched for CD4^+^ cells using EasySep Human CD4^+^ T Cell Isolation Kit (Cat. No. 19052). DNA and RNA were co-extracted using the AllPrep Mini (Cat. No. 80204) from the same CD4^+^ cell sample. RNA was used for total polyadenylated cDNA generation using dT20 primer and reverse transcription was performed with Thermo Scientific SuperScript IV First-Strand Synthesis (Cat. No. 18091150). Resulting HIV-cDNA levels were quantified using BIO-RAD QX200 droplet digital PCR using the following HIV-specific primer and probe sets targeting two regions of the viral transcriptome: HXB2 coordinates 684-810 for unspliced HIV mRNA (Forward primer: 5’-TCTCGACGCAGGACTCG-3’, reverse primer 5’-TACTGACGCTCTCGCACC-3’, and probe 5’-/56-FAM/CTCTCTCCT/ZEN/TCTAGCCTC/31ABkFQ/−3’); HXB2 coordinates 9435-9525 for total polyadenylated viral RNA with forward primer 5’-GGGACTTTCCGCTGGG-3’, reverse primer 5’-AGCAGCTGCTTATATGCAG-3’, and probe 5’-/56-FAM/TGAGGGCTC/ZEN/GCCACTCC/3IABkFQ/−3’. DdPCR data analyses were performed using the BIO-RAD QuantaSoft software suite.

### Tat/Rev induced limiting dilution assay

To estimate the size of the latent reservoir capable of reactivation, we performed the Tat/Rev Induced Limiting Dilution Assay (TILDA)^71^. 10 x 106  PBMCs from study participants were thawed, enriched for CD4^+^ T cells, resuspended at 2 × 10^6^ cells/ml in R10 medium containing antiretrovirals (200 nM emtricitabine and 200 nM dolutegravir) and rested for 2-6 hours at 37 °C, 5% CO2 in a 24-well plate (1 mL/plate). CD4^+^ T cells were stimulated for 12 h with 100 ng/mL PMA and 1 μg/ml ionomycin (both from Sigma). After stimulation, cells were washed in R10, counted and serially diluted to 18 × 10^6^ cells/mL, 9 × 10^6^ cells/ml, 3 × 10^6^ cells/mL and 1 × 10^6^ cells/mL in PBS/FBS 10%. 1 μl from each cell suspension was distributed to 24 wells of a 384-well plate containing 5 μl of 2 × reaction buffer from the SuperScript III Platinum One-Step qRT-PCR Kit (Life Technologies) corresponding to 18,000, 9000, 3000 and 1000 cells per well. Pre-amplification was carried out by adding 5 μl of a PCR mix containing 0.2 μl Superscript III Platinum Taq (Life Technologies, cat # 11-745-100), 0.1 μl RNase inhibitor (Life Technologies, cat # AM2696), 0.125 μl of each primer (tat1.4 and rev both at 20 μM), 2.2 μl Tris–EDTA (TE) buffer and 2.25 μl H2O to each well (final reaction volume = 11 μl). The sequences of the oligonucleotides are as follows: tat1.4: 5′-TGG CAG GAA GAA GCG GAG A-3′; rev: 5′-GGA TCT GTC TCT GTC TCT CTC TCC ACC-3′. Pre-amplification was carried out using the following steps: reverse transcription at 50 °C for 15 min, denaturation at 95 °C for 2 min, 24 cycles of amplification (95 °C 15 s, 60 °C 4 min) on a C1000 Touch PCR instrument (BioRad). This reaction was performed by adding 5 μl of the TaqMan Fast Advanced Master Mix (Invitrogen, cat # 4444556), 0.2 μl of each primer (tat2 and rev, both at a working concentration of 20 μM), 0.2 μl of the probe MSHIV FamZen at 5 μM and 3.4 μl H2O to each well (final reaction volume = 10 μl). Sequence of tat2 and the HIV probe are as follows: tat2: 5′- ACA GTC AGA CTC ATC AAG TTT CTC TAT CAA AGC A −3′. Probe HIV: 5′-/56-FAM/TTC CTT CGG /ZEN/GCC TGT CGG GTC CC/3IABkFQ/−3′. All primers and probes were synthesized by IDT. The real-time PCR reaction was carried out in a QuantStudio 6 Flex (ThermoFisher) using the following program: Preincubation 95 °C for 20 s, 45 cycles of 95 °C 1 s and 60 °C 20 s. Positive wells at each dilution were counted and the maximum likelihood method was used to calculate the frequency of cells with inducible HIV msRNA (http://bioinf.wehi.edu.au/software/elda).

### HIV gp120 enzyme-linked immunosorbent assay (ELISA)

96 well EIA/RIA clear flat bottom polystyrene high bind microplates (Corning) were coated with 1 μg/mL of recombinant YU-2 gp120 provided by Dr. Mascola (VRC, NIH) protein in phosphate-buffered saline (PBS) overnight at 4 °C. Plates were blocked with B3T buffer (30 mM NaCl, 10 mM Tris-HCl, 0.2 mM EDTA, 0.66% fetal bovine serum, 0.4% bovine albumin, 0.014% Tween 20, 0.004% thimersol) and incubated with 5-fold serial dilutions of heat-inactivated plasma starting at a dilution of 1:100. After incubation with peroxidase-conjugated goat anti-human IgG antibody (Jackson ImmunoResearch), SureBlue TMB substrate (Kirkegaard & Perry Laboratories) was added, and plates were read at 450 nm. All incubations were for 1 h at 37 °C, and all volumes were 100 ml, except for blocking, which was 200 ml. The plates were washed 6 times between incubations with PBS-T (PBS with 0.1% Tween 20). Each plasma sample was run one time. After background subtraction, results were plotted and fit by nonlinear regression using the sigmoidal dose-response (variable slope) model in GraphPad Prism.

### Reporting summary

Further information on research design is available in the Nature Research Reporting Summary linked to this article.

## Supplementary information

Supplementary Information

Peer Review File

Reporting Summary

## Data Availability

All data that support the findings of this study are available in the manuscript, Figures and Supplementary Figures. The gene counts of the bulk RNA-seq data can be found at https://github.com/abcwcm/CovaxxHIV10.5281/zenodo.6360535. Gene counts are the least processed data that do not contain the actual genetic sequencing information from study participants (which is Protected Health Information). Source data for all figures are provided with this paper. Source data are provided with this paper.

## Source data

Source Data

## Code availability

The code for the analysis and visualization of the bulk RNA-seq data can be found at https://github.com/abcwcm/CovaxxHIV10.5281/zenodo.6360535. All other algorithm or software central to this paper have been reported in published research papers, as cited in the references. Raw reads were quality checked with FastQC v0.11.7 (http://www.bioinformatics.babraham.ac.uk/projects/fastqc/), and adapters were trimmed using Trim Galore v0.6.7 (http://www.bioinformatics.babraham.ac.uk/projects/trim_galore/). Reads were aligned to the human reference genome (GRCh38.p12) using STAR v2.6.0c^63^ with default parameters. Gene abundances were calculated with featureCounts v1.6.2^64^ using composite gene models from Gencode release 28^65^. Principle component analysis was performed using the plotPCA function from DESeq2 v1.32.0^66^, after removing study participant-specific effects with limma v3.48.0 removeBatchEffect^67^. Differentially expressed genes were determined with DESeq2 v1.32.0 using Wald tests (q < 0.05) with a two-factor model incorporating study participant. Gene set enrichment analysis was performed using fgsea v1.18.0^68^; genes were ordered between treated and untreated cells by the DESeq2 Wald statistic. Gene sets were retrieved from the Broad Institute’s MSigDB collections^69,70^.
