# Supplementary material for: SARS CoV-2 mRNA vaccination exposes latent HIV to Nef-specific CD8+ T-cells
Source: Nat Commun. 2022 Aug 19;13:4888. doi: 10.1038/s41467-022-32376-z (PMC9389512; doi:10.1038/s41467-022-32376-z)
Supplement: Supplementary file 1 — Supplementary Information [file 41467_2022_32376_MOESM1_ESM.pdf]

Supplementary Information

SARS CoV-2 mRNA vaccination exposes latent HIV to Nef-specific CD8$^+$ T-cells

Eva M. Stevenson[1*], Sandra Terry[1*], Dennis Copertino[1*], Louise Leyre[1,2], Ali Danesh[1], Jared Weiler[1], Adam R. Ward[1], Pragya Khadka[1], Kevin Bernard[1], Itzayana G. Miller[1], Grant B. Ellsworth[1], Carrie D. Johnston[1], Eli J. Finkelsztein[1], Paul Zumbo[8], Doron Betel[1,8], Friederike Dündar[8,9], Evan McNeil[1], Maggie C. Duncan[3,4], Hope R. Lapointe[4], Sarah Speckmaier[4], Nadia Moran-Garcia[4], Michelle Premazzi Papa[5], Samuel Nicholes[5], Carissa J. Stover[5], Rebecca M. Lynch[5], Marina Caskey[6], Christian Gaebler[6], Tae-Wook Chun[7], Alberto Bosque[5], Timothy J. Wilkin[1], Guinevere Q. Lee[1], Zabrina L. Brumme[3,4], R. Brad Jones[1,2]

[1] Department of Medicine, Weill Cornell Medical College, New York, NY, USA.
[2] Immunology and Microbial Pathogenesis Program, Weill Cornell Graduate School of Medical Sciences, New York, NY.
[3] Faculty of Health Sciences, Simon Fraser University, Burnaby, BC, Canada.
[4] British Columbia Centre for Excellence in HIV/AIDS, Vancouver, BC, Canada
[5] Dept of Microbiology Immunology and Tropical Medicine, The George Washington University, Washington, DC, USA.
[6] Laboratory of Molecular Immunology, The Rockefeller University, New York, NY, USA.
[7] Laboratory of Immunoregulation, National Institute of Allergy and Infectious Diseases (NIAID, NIH, Bethesda, MD, USA.
[8]Applied Bioinformatics Core, Weill Cornell Medical College, New York, NY, USA
[9]Department of Physiology and Biophysics, Weill Cornell Medical College, New York, NY, USA

Supplementary Table 1. Participant demographic and clinical data, ex vivo latency reversal.

Participant ID	Age at Enrollment (years)	Race	Ethnicity	Sex	Gender Identity	COVID Vaccine Received	Anti-Spike Serology at Entry	Year of HIV Diagnosis	Year started multi-agent ART	Most recent CD4 (cells/ul)	Nadir CD4 if known (cells/uL)	Current ART	Sample Date
OM5011	46	White	Caucasian	Male	Man	NA	NA	2005	2008	602	340	ABC/DTG/3TC	10/7/21
OM5258	62	White	Caucasian	Male	Man	NA	NA	1989	2002	581	240	ABC/DTG/3TC	8/1/17
OM5334	29	White	NA	Male	Man	NA	NA	2013	2013	840	460	TAF/FTC/c/EVG/RPV	4/18/18
WWH-B005	51	White	Caucasian	Male	Man	NA	NA	1995	2005	311	176	FTC/TAF/RIL/DRV/c/DTG	12/4/17
WWH-B029	35	White	Caucasian	Male	Man	NA	NA	2018	2018	287	Unknown	TAF/FTC/BIC	4/18/18
WWH-B032	62	African-American	Not Hispanic or Latino	Male	Man	NA	NA	1983	2005	212	181	TDF/FTC/DRV/r/ETR	4/30/18

Supplementary Table 2. Participant demographic and clinical data, booster cohort.

Participant ID	Age at Enrollment (years)	Race	Ethnicity	Sex	Gender Identity	COVID Booster Vaccine Received	Year of HIV Diagnosis	Year started multi-agent ART	Most recent CD4 (cells/ul)	Nadir CD4 (cells/uL)	Current ART
12	29	White	Not Hispanic or Latinx	Male	Man	mRNA 1273	2016	2016	760	640	TAF/FTC/c/EVG
20	34	White	Not Hispanic or Latinx	Male	Man	BNT162b2	2010	2012	1066	480	RPV/DTG
21	57	White	Hispanic or Latinx	Female	Woman	mRNA 1273	1988	1996	493	30	TAF/FTC/BIC
23	41	unknown	Hispanic or Latinx	Male	Man	BNT162b2	2000	2005	620	300	ABC/3TC/DTG
24	40	White	Hispanic or Latinx	Male	Man	BNT162b2	2006	2018	720	unknown	TAF/FTC/BIC
25	43	White	Hispanic or Latinx	Male	Man	BNT162b2	2017	2017	864	584	RPV/DTG
27	58	Black or African American	Not Hispanic or Latinx	Male	Man	mRNA 1273	1997	2005	699	300	BIC/FTC/TAF
28	63	Black or African American	Not Hispanic or Latinx	Female	Woman	BNT162b2	1991	2002	1895	94	RPV/DTG

Supplementary Table 3. Participant demographic and clinical data, no vaccine cohort

Record ID	Age at Enrollment (years)	Race	Ethnicity	Sex	Gender Identity	COVID Vaccine Received	Anti-Spike Serology at Entry	Year of HIV Diagnosis	Year started multi-agent ART	Most recent CD4 (cells/ul)	Nadir CD4 if known (cells/uL)	Current ART
B633	50	White	Not Hispanic or Latinx	Male	NA	NA	NA	2013	2013	1198	unknown	DTG/RPV
B667	43	Multiple Race	Hispanic or Latinx	Male	NA	NA	NA	2010	2010	851	>500	BIC/FTC/TAF
B673	65	Multiple Race	Not Hispanic or Latinx	Male	NA	NA	NA	1989	2002	510	200	FTC/TAF/ATV/COBI
9A02/B317	58	Black or African American	Not Hispanic or Latinx	Female	NA	NA	NA	1996	2003	1351	1000	EVG/COBI/FTC/TAF
9A06/B285	50	Black or African American	Not Hispanic or Latinx	Male	NA	NA	NA	2012	2012	465	100	EFV/FTC/TDF
9B02/B342	48	Black or African American	Not Hispanic or Latinx	Male	NA	NA	NA	2000	2000	398	400	FTC/RPV/TAF
9B05/B352	54	Black or African American	Not Hispanic or Latinx	Male	NA	NA	NA	1991	2000	575	<10	RAL/TDF/RTV/SQV

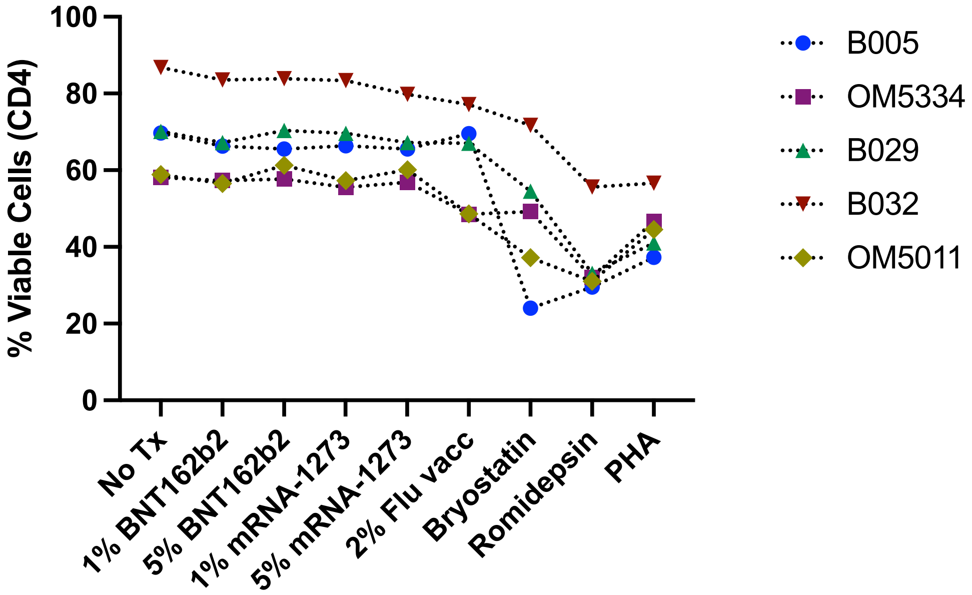

Supplementary Fig. 1. Viability of $CD4^+$ T-cells at conclusion of ex vivo latency reversal experiments. Shown are flow cytometry results where % viable cells were determined based on LIVE/DEAD™ fixable aqua dead cell staining.

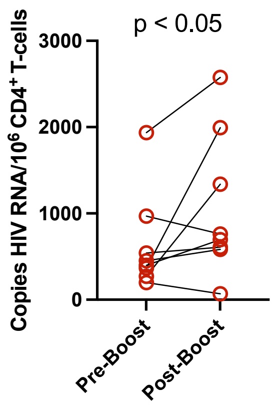

Supplementary Fig. 2. Increases in cell-associated HIV RNA following second SARS CoV-2 mRNA vaccine boosters. Shown are mean levels of unspliced cell-associated HIV RNA (from technical triplicates), assessed prior to and within days (median 4, range 3-5 days) of receiving mRNA vaccine boosts. The P value of 0.0470 was calculated by Paired t test (one-tailed).

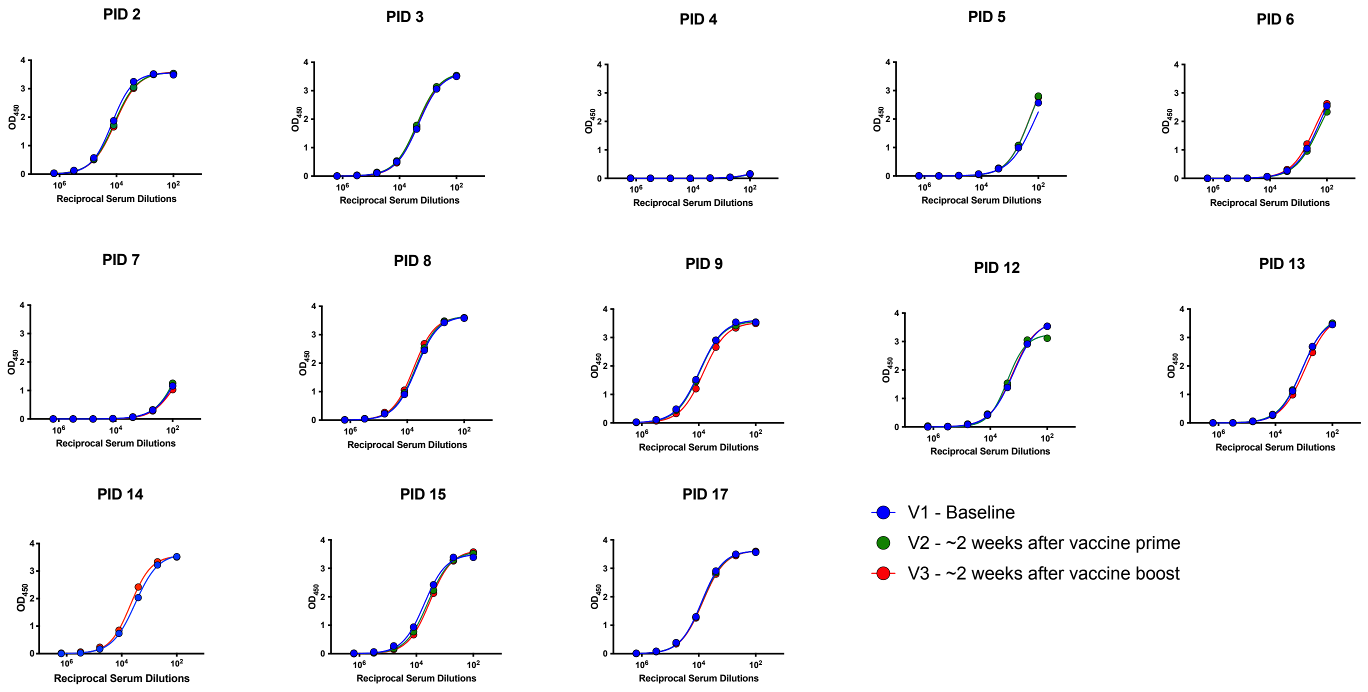

Supplementary Fig. 3. No detectable changes in anti-HIV gp120 antibody titers following COVID vaccine dose 1 or dose 2. Shown are antibody binding results to rgp120 YU-2 graphing optical density (OD_{450}) at the indicated plasma dilutions for each participant. Timepoints for graphed samples are baseline (V1), and ~2 weeks after vaccine dose 1 (V2) or vaccine dose 2 (V3). After background subtraction, results were plotted and fit by nonlinear regression using the sigmoidal dose-response (variable slope) model in GraphPad Prism.

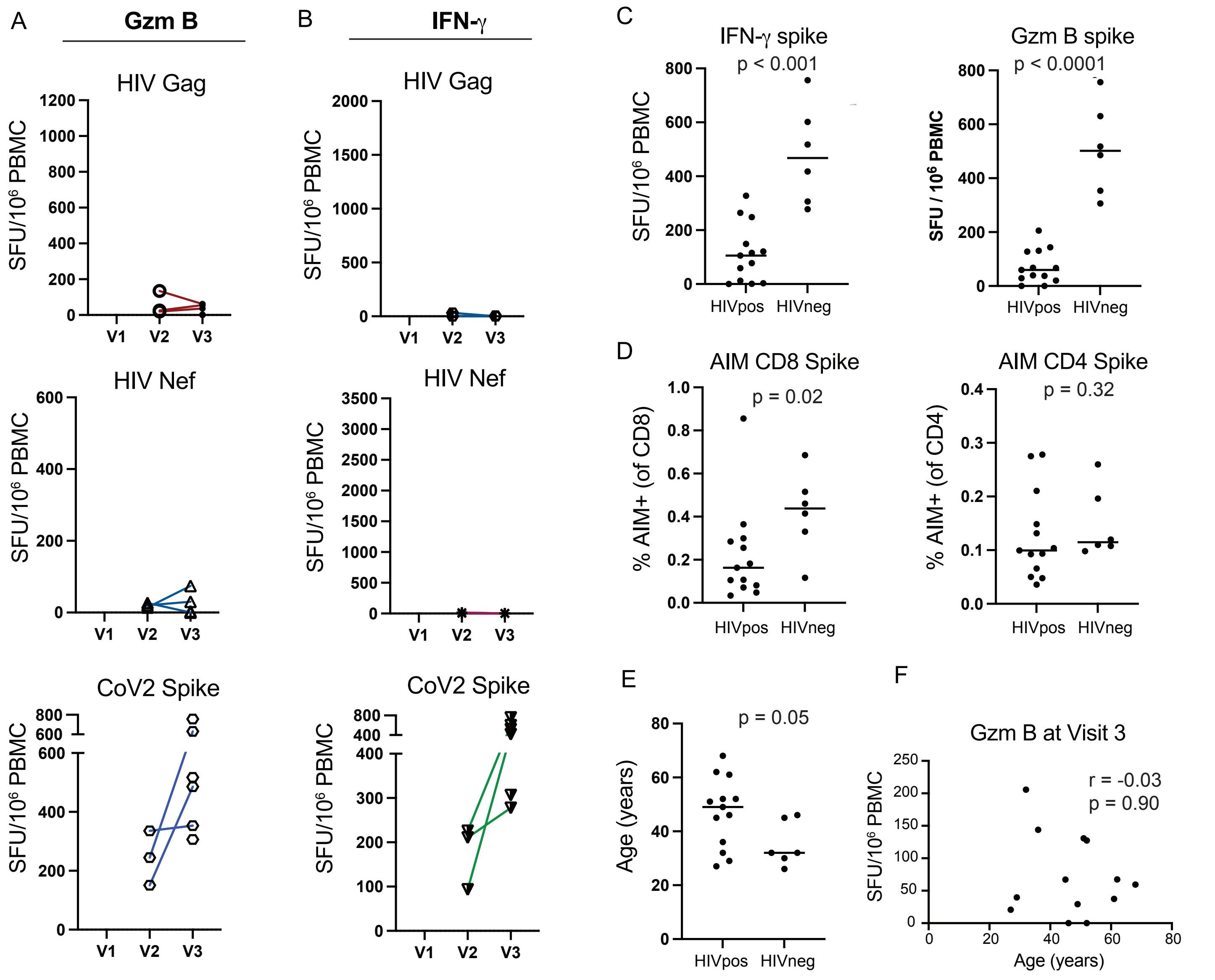

Supplementary Fig. 4. Comparisons with T-cell responses in an HIV-negative cohort. A – Gzm B, and **B** – IFN-γ ELISPOT responses, plotted on same axes as HIV-positive cohort in **Fig. 4**. The lack of induction of HIV-specific responses rules out CoV-2 cross-reactivity as a driver of the increases in Nef- and Rev-specific T-cell responses observed at the first vaccine dose in the HIV-positive cohort. **C & D**. Comparisons of CoV-2-Spike-specific T-cell responses at V3 (~2 weeks post second dose) between an HIV-positive versus HIV-negative cohort. **C** – ELISPOT results, **D** – Activation induced marker (AIM) results, two-tailed Mann-Whitney tests. Exact P value for IFN-γ ELISPOT Spike – P = 0.0003, and given as P < 0.0001 as an Exact value by the Prism software for Gzm-B ELISPOT Spike. **E**. Comparisons of ages across HIV-positive versus HIV-negative cohorts show that

the former tended to be older, approaching statistical significance (two-tailed Mann-Whitney test). **F**. No significant correlations were observed within the HIV-positive cohort between age and CoV-2-specific T-cell responses by any measure tested, at V3; shown are results for granzyme B ELISPOT – two-tailed Spearman's correlation test. For each of **A – E**: n = 13 for HIV-positive cohort at V1 and V3 and n = 12 at V2 (PID 14 missed V2); n = 3 at V2 and n = 6 at V3 for HIV-negative cohort (three of the V3 donors did not have a visit 2).

Our study provided the additional opportunity to compare vaccine-induced Spike-specific T-cell responses with a cohort of HIV-negative donors collected at similar timepoints following vaccine dose 1 (n = 3) and dose 2 (n = 6) (median, range: 22, 18-27 days after dose 2). Following second vaccine doses, we observed significantly higher magnitudes of SARS-CoV2-Spike-specific T-cells by both IFN-γ and Gzm B ELISPOT (IFN-γ mean, 114 SFU/10^6 PBMCs in HIVpos versus 480 SFU/10^6 PBMCs in HIVneg – $p < 0.001$; Gzm B mean, 71 SFU/10^6 PBMCs in HIVpos versus 508 SFU/10^6 PBMCs in HIVneg – $p < 0.0001$, **Supplementary Fig. 4C**). By AIM assay, we also observed higher frequency CD8$^+$ T-cell responses in HIVneg versus HIVpos individuals (mean 0.42 % AIM+ versus 0.11 % AIM+, p = 0.02), whereas a significant difference was not observed in CD4+ T-cell responses (p = 0.32) (**Supplementary Fig. 4D**). A caveat to this side-observation of our study is that the HIVpos and HIVneg cohorts were not age matched, with a younger HIVneg cohort (p = 0.05, **Supplementary Fig. 4E**). However, we note that we observed a lack of correlations between age and any of our T-cell measures following second vaccine doses – shown for gzm-B ELISPOT in **Supplementary Fig. 4F**. Additionally, the cohorts were not vaccine matched. The HIVneg cohort had more individuals that received the mRNA1273 vaccine (**Table 1 and Supplementary Table 6).**

Supplementary Table 4. Spearman correlations between changes in HIV RNA (V3 / V1) and T-cell responses measured by ELISPOT. Correlation results were corrected post hoc for multiple comparisons by the false discovery rate method of Benjamini and Hochberg using the SAS MULTTEST procedure (FDR option)

				Gag	Env	Pol	Nef	Tat	Rev	Vif/Vpr/Vpu	CMV-pp65	CoV-2 Spike
**Granzyme B**	5' RNA (unspliced)	V1	Spearman r	-0.090	-0.250	-0.220	-0.220	0.070	-0.050	0.340	-0.050	0.080
			Spearman p	0.780	0.410	0.460	0.470	0.820	0.870	0.250	0.870	0.800
			FDR adjusted p	0.890	0.970	0.460	0.470	0.820	0.870	0.570	0.870	0.800
		V2	Spearman r	-0.330	-0.014	-0.260	-0.510	-0.120	-0.270	-0.090	-0.110	-0.320
			Spearman p	0.290	0.970	0.410	0.090	0.710	0.390	0.780	0.730	0.270
			FDR adjusted p	0.870	0.970	0.460	0.270	0.820	0.870	0.780	0.870	0.405
		V3	Spearman r	0.040	-0.080	-0.370	-0.370	-0.210	-0.090	-0.260	-0.200	0.420
			Spearman p	0.890	0.790	0.210	0.210	0.480	0.760	0.380	0.530	0.150
			FDR adjusted p	0.890	0.970	0.460	0.315	0.820	0.870	0.570	0.870	0.405
	3' RNA (total)	V1	Spearman r	-0.060	-0.240	-0.350	-0.430	-0.060	-0.140	-0.500	-0.220	0.070
			Spearman p	0.850	0.450	0.270	0.160	0.340	0.650	0.100	0.470	0.830
			FDR adjusted p	0.870	0.450	0.525	0.160	0.390	0.650	0.300	0.470	0.970
		V2	Spearman r	-0.050	-0.310	-0.290	**-0.730**	-0.400	-0.270	-0.170	-0.310	-0.020
			Spearman p	0.870	0.330	0.350	**0.006**	0.194	0.390	0.600	0.320	0.970
			FDR adjusted p	0.870	0.450	0.525	**0.017**	0.390	0.585	0.600	0.470	0.970
		V3	Spearman r	-0.120	-0.240	-0.200	-0.540	-0.270	-0.610	-0.340	-0.310	-0.010
			Spearman p	0.700	0.450	0.530	0.070	0.390	0.040	0.270	0.320	0.970
			FDR adjusted p	0.870	0.450	0.530	0.105	0.390	0.120	0.405	0.470	0.970
**IFN-γ**	5' RNA (unspliced)	V1	Spearman r	-0.020	-0.290	0.070	-0.120	-0.400	-0.170	-0.170	-0.190	0.550
			Spearman p	0.960	0.350	0.830	0.720	0.200	0.590	0.030	0.540	0.310
			FDR adjusted p	0.960	0.525	0.970	1.000	0.260	0.885	0.090	0.570	0.465
		V2	Spearman r	0.100	-0.300	-0.010	0.000	-0.440	0.000	-0.150	-0.280	0.110
			Spearman p	0.770	0.340	0.970	1.000	0.150	1.000	0.650	0.380	0.740
			FDR adjusted p	0.960	0.525	0.970	1.000	0.260	1.000	0.975	0.570	0.740
		V3	Spearman r	0.150	-0.150	0.150	0.010	-0.350	-0.390	0.000	-0.170	0.500
			Spearman p	0.650	0.630	0.650	0.970	0.260	0.210	0.990	0.570	0.100
			FDR adjusted p	0.960	0.630	0.970	1.000	0.260	0.630	0.990	0.570	0.300
	3' RNA (total)	V1	Spearman r	-0.020	-0.300	0.070	-0.012	-0.400	-0.170	-0.090	0.400	0.120
			Spearman p	0.960	0.350	0.830	0.720	0.200	0.590	0.780	0.210	0.740
			FDR adjusted p	0.960	0.525	0.970	1.000	0.260	0.885	0.960	0.620	0.740
		V2	Spearman r	0.100	-0.300	-0.010	0.000	-0.440	0.000	-0.020	0.220	0.280
			Spearman p	0.770	0.340	0.970	1.000	0.150	1.000	0.960	0.500	0.370
			FDR adjusted p	0.960	0.525	0.970	1.000	0.260	1.000	0.960	0.620	0.555
		V3	Spearman r	0.150	-0.150	0.150	0.010	-0.350	-0.390	0.340	0.160	0.500
			Spearman p	0.650	0.630	0.650	0.970	0.260	0.210	0.270	0.620	0.100
			FDR adjusted p	0.960	0.630	0.970	1.000	0.260	0.630	0.810	0.620	0.300

Supplementary Table 5. Spearman correlations between fold changes in HIV RNA (V3 / V1) and T-cell responses measured by AIM assay. Correlation results were corrected post hoc for multiple comparisons by the false discovery rate method of Benjamini and Hochberg using the SAS MULTTEST procedure (FDR option)

			**CD8**				**CD4**			
			HIV-Gag	HIV-Nef	CMV-pp65	CoV-2 Spike	HIV-Gag	HIV-Nef	CMV-pp65	CoV-2 Spike
5' RNA (unspliced)	V1	Spearman r	0.080	-0.390	-0.670	-0.660	-0.250	-0.590	-0.360	-0.260
		Spearman p	0.790	0.190	0.020	0.020	0.400	0.040	0.230	0.380
		FDR adjusted p	1.000	0.285	0.060	0.060	0.675	0.120	0.435	0.520
	V2	Spearman r	0.000	-0.510	-0.170	-0.380	0.240	-0.220	-0.200	0.240
		Spearman p	1.000	0.090	0.600	0.220	0.450	0.500	0.520	0.440
		FDR adjusted p	1.000	0.270	0.600	0.330	0.675	0.520	0.520	0.520
	V3	Spearman r	0.200	0.000	-0.250	0.190	0.110	-0.200	-0.320	0.200
		Spearman p	0.520	1.000	0.410	0.530	0.730	0.520	0.290	0.520
		FDR adjusted p	1.000	1.000	0.600	0.530	0.730	0.520	0.435	0.520
3' RNA (total)	V1	Spearman r	-0.300	-0.360	-0.170	-0.020	-0.530	-0.500	0.150	0.240
		Spearman p	0.340	0.240	0.590	0.960	0.080	0.100	0.650	0.460
		FDR adjusted p	0.510	0.240	0.600	0.960	0.240	0.150	0.910	0.690
	V2	Spearman r	-0.360	**-0.760**	-0.170	-0.480	-0.350	-0.660	-0.110	-0.310
		Spearman p	0.250	**0.006**	0.600	0.110	0.260	0.020	0.740	0.330
		FDR adjusted p	0.510	**0.017**	0.600	0.195	0.260	0.060	0.910	0.690
	V3	Spearman r	-0.170	-0.540	-0.270	-0.460	-0.430	-0.430	-0.040	0.020
		Spearman p	0.600	0.070	0.390	0.130	0.160	0.160	0.910	0.960
		FDR adjusted p	0.600	0.105	0.600	0.195	0.240	0.160	0.910	0.960

Supplementary Table 6. Participant demographic and clinical data in the Rockefeller University cohort of people without HIV.

Record ID	Age at Enrollment (years)	Race	Ethnicity	Sex	COVID Vaccine Received
C012	30	White	non-Hispanic	F	mRNA 1273
C016	32	White	Hispanic	M	mRNA 1273
C037	46	White	Hispanic	F	BNT162b2
C043	26	White	non-Hispanic	F	mRNA 1273
C044	32	White	non-Hispanic	F	BNT162b2
C049	45	White	Hispanic	M	BNT162b2

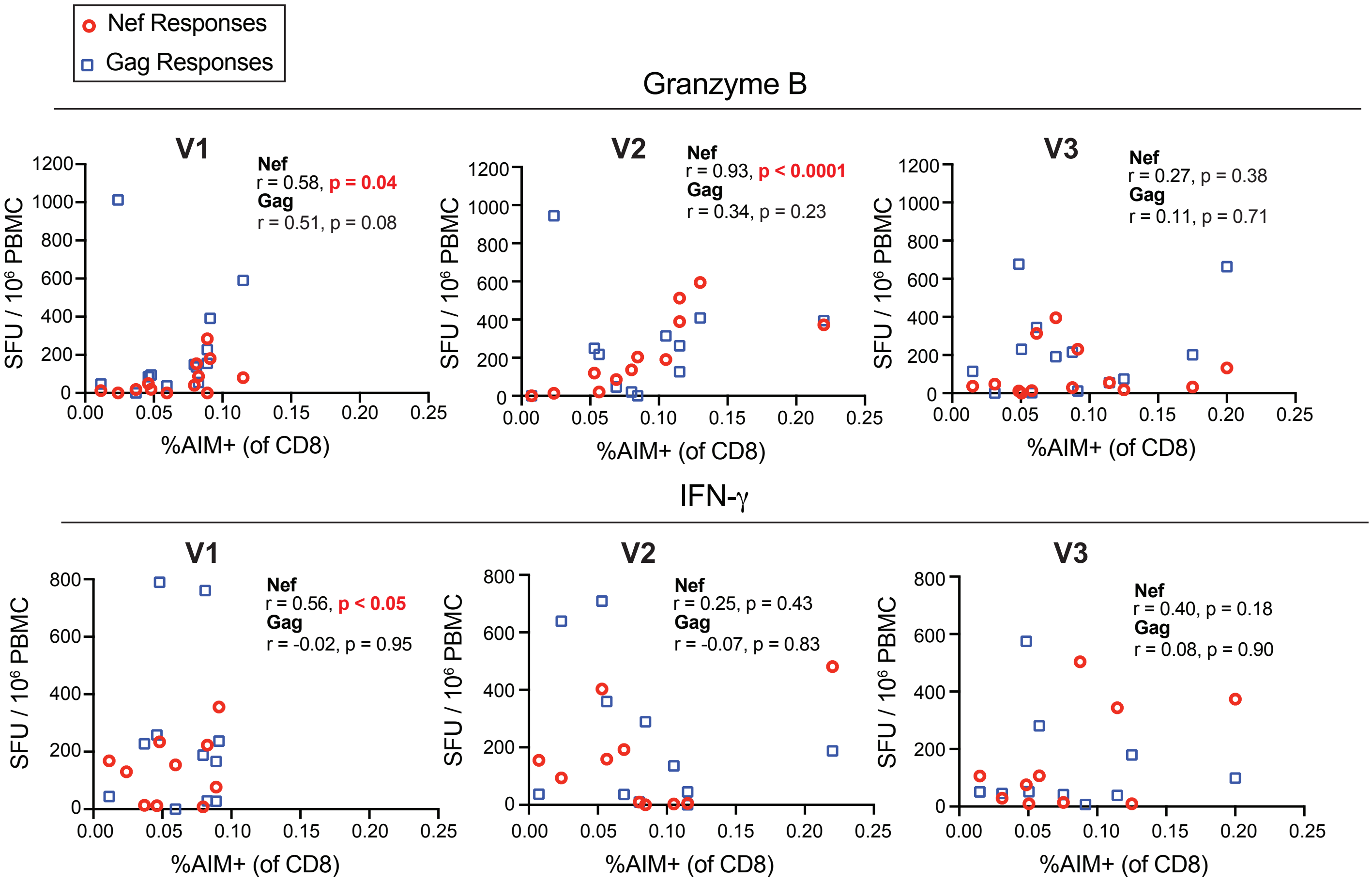

Supplementary Fig. 5. Correlations between T-cell responses as measured by AIM and ELISPOT. Shown are Spearman correlations for Nef- or Gag-specific $CD8^+$ T-cell responses as measured by AIM assay with IFN-γ or Granzyme B ELISPOT. For the correlation with Nef Gzm-B responses at V2 the P value of < 0.0001 is reported as Exact by the Prism software. The exact P value for Nef IFN-γ responses at V1 is 0.0488.

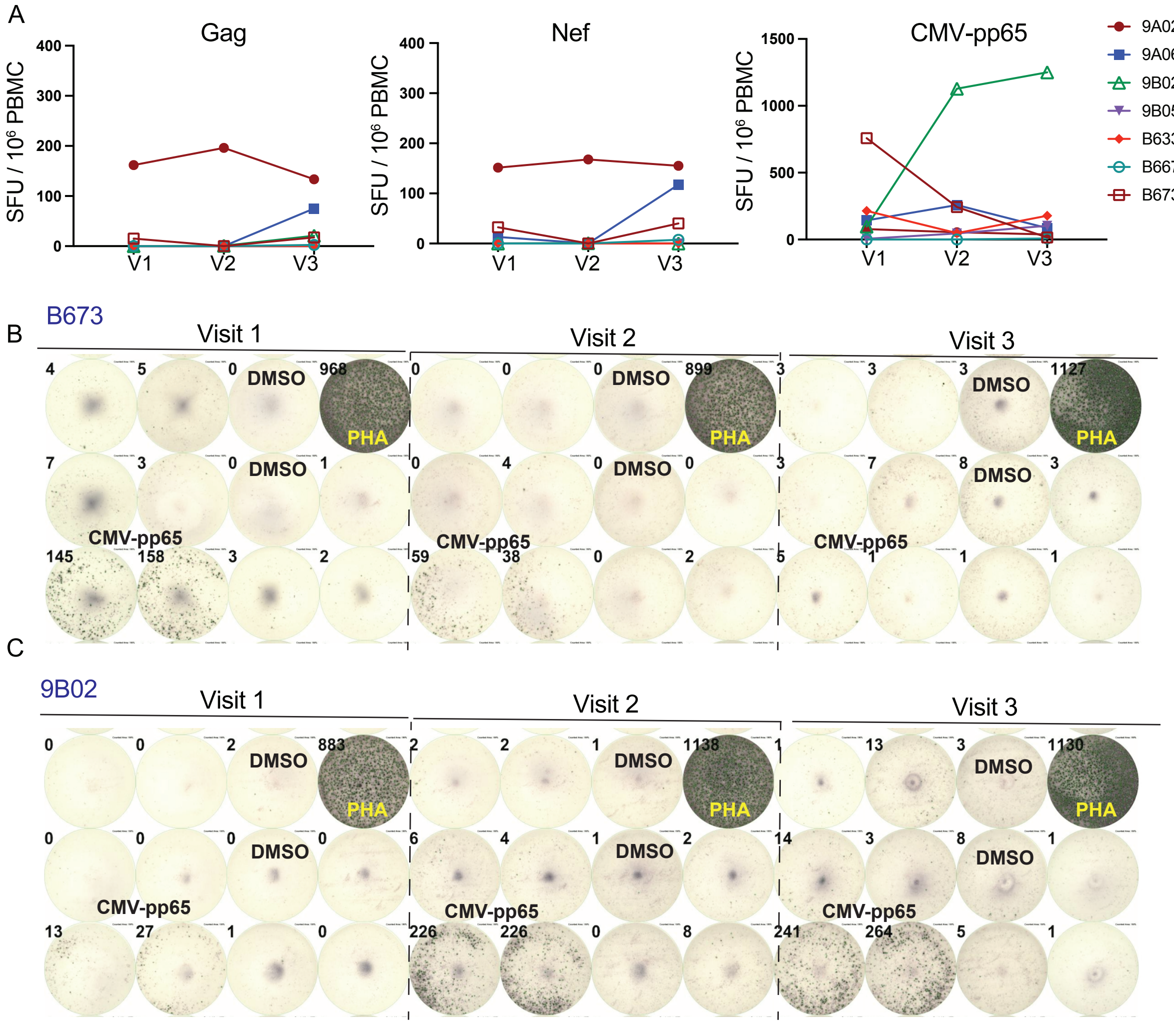

Supplementary Fig. 6. Granzyme B T-cell response dynamics in a cohort not receiving mRNA vaccine. A. Summary granzyme B ELISPOT data for a cohort of PWH that did not receive an mRNA vaccine over the course of study visits (clinical and demographic data in **Supplementary Table 3**). Median and range times between V1 – V2 = 21, 16-25 days and V2 – V3 = 18, 13-25 days. **B & C.** Granzyme B ELISPOTs for two donors – one showing a decrease in CMV-pp65-specific responses over the study (B673) and the other showing an increase in CMV-pp65 responses over the study (9B02).

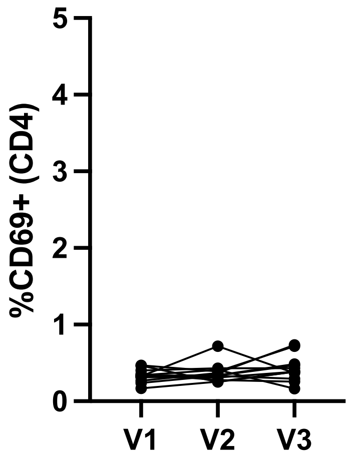

Supplementary Fig. 7. Lack of general induction of CD69 expression (activation) following CoV-2 mRNA vaccinations. Shown are flow cytometry data gating on viable $CD4^+$ T-cells on the main New York cohort (**Table 1**).
