# Supplementary material for: SARS CoV-2 mRNA vaccination exposes latent HIV to Nef-specific CD8+ T-cells
Source: Nat Commun. 2022 Aug 19;13:4888. doi: 10.1038/s41467-022-32376-z (PMC9389512; doi:10.1038/s41467-022-32376-z)
Supplement: Supplementary file 3 — Reporting Summary [file 41467_2022_32376_MOESM3_ESM.pdf]

nature portfolio

Corresponding author(s): Dr. R. Brad Jones

Last updated by author(s): June 5, 2022

Reporting Summary

Nature Portfolio wishes to improve the reproducibility of the work that we publish. This form provides structure for consistency and transparency in reporting. For further information on Nature Portfolio policies, see our Editorial Policies and the Editorial Policy Checklist.

Statistics

For all statistical analyses, confirm that the following items are present in the figure legend, table legend, main text, or Methods section.

n/a	Confirmed	
☐	☒	The exact sample size (*n*) for each experimental group/condition, given as a discrete number and unit of measurement
☐	☒	A statement on whether measurements were taken from distinct samples or whether the same sample was measured repeatedly
☐	☒	The statistical test(s) used AND whether they are one- or two-sided<br>*Only common tests should be described solely by name; describe more complex techniques in the Methods section.*
☐	☒	A description of all covariates tested
☐	☒	A description of any assumptions or corrections, such as tests of normality and adjustment for multiple comparisons
☐	☒	A full description of the statistical parameters including central tendency (e.g. means) or other basic estimates (e.g. regression coefficient) AND variation (e.g. standard deviation) or associated estimates of uncertainty (e.g. confidence intervals)
☐	☒	For null hypothesis testing, the test statistic (e.g. *F*, *t*, *r*) with confidence intervals, effect sizes, degrees of freedom and *P* value noted<br>*Give P values as exact values whenever suitable.*
☒	☐	For Bayesian analysis, information on the choice of priors and Markov chain Monte Carlo settings
☒	☐	For hierarchical and complex designs, identification of the appropriate level for tests and full reporting of outcomes
☐	☒	Estimates of effect sizes (e.g. Cohen's *d*, Pearson's *r*), indicating how they were calculated

Our web collection on statistics for biologists contains articles on many of the points above.

Software and code

Policy information about availability of computer code

Data collection	ddPCR data was collected using QuantaSoft (BioRad Laboratories, Inc., version 1.7.4.0917), flow cytometry data was collected using Attune NxT software (thermofisher scientific version 3.1.2)
Data analysis	ddPCR data was collected using QuantaSoft (BioRad Laboratories, Inc., version 1.7.4.0917), flow cytometry data was analyzed using FlowJo software (Treestar, version 10.7.1). Statistical analysis was performed in Prism 9 for mac OS. RNA seq analysis: Please see last page

For manuscripts utilizing custom algorithms or software that are central to the research but not yet described in published literature, software must be made available to editors and reviewers. We strongly encourage code deposition in a community repository (e.g. GitHub). See the Nature Portfolio guidelines for submitting code & software for further information.

Data

Policy information about availability of data

All manuscripts must include a data availability statement. This statement should provide the following information, where applicable:

- Accession codes, unique identifiers, or web links for publicly available datasets
- A description of any restrictions on data availability
- For clinical datasets or third party data, please ensure that the statement adheres to our policy

All data that support the findings of this study are available in the manuscript, Figures and Extended Data Figures. The gene counts and code for the analysis and visualization of the bulk RNA-seq data can be found at https://github.com/abcwcm/CovaxxHIV DOI: 105281/zenodo.6360535. Additional information is available from the corresponding authors upon request.

Field-specific reporting

Please select the one below that is the best fit for your research. If you are not sure, read the appropriate sections before making your selection.

[x] Life sciences   [ ] Behavioural & social sciences   [ ] Ecological, evolutionary & environmental sciences

For a reference copy of the document with all sections, see nature.com/documents/nr-reporting-summary-flat.pdf

Life sciences study design

All studies must disclose on these points even when the disclosure is negative.

Sample size	Our sample sizes of n=13 (New York) and n=16 (Vancouver) were determined by the participants who we were able to enroll in our study based on our inclusion and exclusion criteria. The suitability of the sample size to support our conclusions is demonstrated by the significant p values of the corresponding statistical tests.
Data exclusions	New York cohort: No data exclusions were performed. The V2 time point includes only 12 of 13 participants since one individual missed visit 2. Vancouver cohort: Results from one individual in an initial set of 16 participants were excluded on the basis of extremely poor viability and yield from the cryopreserved sample (9.9% viable, only 2,300 viable CD8+ events in flow cytometry file).
Replication	All ELISPOT, AIM+, ddPCR assays were performed as technical replicates, and we observed good agreement between these - see ELISPOT examples in Figure 3. ELISAs were assessed with single wells for each dilution of plasma and each sample was run in a 7-point dilution series. We did not have sufficient sample availability to perform multiple experiments independently therefore we used technical replicates.
Randomization	Not applicable. Division of participants/samples into different conditions was not necessary to address the research question in this study. A comparison between an HIV-negative and HIV-positive cohort is presented in the extended data as a non-conclusive observation.
Blinding	Not applicable as participants/samples were not divided into different conditions in this study

Reporting for specific materials, systems and methods

We require information from authors about some types of materials, experimental systems and methods used in many studies. Here, indicate whether each material, system or method listed is relevant to your study. If you are not sure if a list item applies to your research, read the appropriate section before selecting a response.

Materials & experimental systems

n/a	Involved in the study	
[ ]	[x]	Antibodies
[x]	[ ]	Eukaryotic cell lines
[x]	[ ]	Palaeontology and archaeology
[x]	[ ]	Animals and other organisms
[ ]	[x]	Human research participants
[x]	[ ]	Clinical data
[x]	[ ]	Dual use research of concern

Methods

n/a	Involved in the study	
[x]	[ ]	ChIP-seq
[ ]	[x]	Flow cytometry
[x]	[ ]	MRI-based neuroimaging

Antibodies

Antibodies used	Flow antibodies: Fluorochrome conjugated antibodies from Biolegend: anti-CD3-Brilliant Violet 785 clone SK7 (cat# 344842), anti-CD8-BV605 clone RPA-T8 (cat# 301040), anti-CD137-APC (4-1BB) clone 4B4-1 (cat # 309810), anti-OX40-Brilliant Violet 711 clone Ber-ACT35 (ACT35) (cat # 350030), anti-CXCR5-AF488 clone J252D4 (cat # 356912), and anti-CD4-AF700 clone A161A1 (cat # 357418), anti-human-CD25-APC/Cy7 clone BC96 (cat#302614); the following from Invitrogen: Anti-CD69-APC-eFluor 780 clone FN50 (cat# 47-0699-42), as well as fixable aqua viability dye (cat # L34966) and anti-HIV-1 core antigen p24-RD-1 clone KC57 (Beckman Coulter cat#6604667).<br>ELISPOT antibodies: IFN-gamma and Granzyme B antibodies from Mabtech Interferon-gamma (coating antibody: clone 1-D1K cat # 3420-2A; biotinylated antibody: clone 7-B6-1) and Granzyme B (coating antibody clone MT28 cat # 3486-2A; biotinylated antibody: clone MT8610) enzyme-linked immune absorbent spot (ELISPOT) assays<br>ELISA antibodies: peroxidase-conjugated goat anti-human IgG antibody (Jackson ImmunoResearch cat#109-035-098). Note that ELISAs also used the following recombinant protein (though not an antibody) recombinant YU-2 gp120 (provided by Dr. Mascola (VRC, NIH).
Validation	Antibodies used for flow cytometry and ELISPOT were purchased from commercial suppliers and had been validated by suppliers and the authors in multiple experiments. Antibodies used for ELISA were obtained from the NIH or purchased from a commercial supplier and had been validated by suppliers and the authors in multiple experiments.

Human research participants

Policy information about studies involving human research participants

Population characteristics	Relevant population characteristics including covariate summary statistics are provided in Table 1 and Extended Data Tables 1, 2, and 5.
Recruitment	Study participants with HIV were recruited at Weill Cornell Medicine's Uptown or Chelsea Clinical Research Site. The Institutional Review Board at Weill Cornell Medicine approved this study (IRB# 21-02023358). Informed consent was obtained from all participants. Inclusion criteria were 18-89 years of age, people living with HIV with sustained HIV suppression for at least one year, HIV viral load <50 copies/mL within 12 months prior to baseline visit and planned receipt of vaccination with an mRNA-based SARS-CoV-2 vaccine. Exclusion criteria included contraindication to receipt of SARS-CoV-2 vaccination, plasma HIV RNA >200 copies/mL within one year prior to the baseline visit, known anemia with a hemoglobin < 10 gm/dL, prior receipt of SARS-CoV-2 vaccination, and discontinuation of ART for 7 or more consecutive days within the prior. Blood samples used in this study were collected at the baseline visit up to 6 weeks prior to planned vaccination, 2 weeks after the first vaccine dose, and 2 weeks after the second vaccine dose. Participants completed a post-vaccine side effects survey after receipt of each dose. Results were recorded and stored in REDCap. Blood was collected via phlebotomy in gold top serum separator tubes (SST) for anti-S SARS-CoV-2 serology, pearl top plasma preparation tubes (PPT) for HIV viral load, or ethylenediaminetetraacetic acid (EDTA) tubes for PBMC processing. HIV viral load and anti-S SARS-CoV-2 serology were performed by the New York-Presbyterian Hospital clinical laboratory.Deidentified samples from adults without HIV were obtained from the Rockefeller University (IRB protocol DRO-1006). Eligible participants were healthy adults with no history of infection with SARS-CoV-2, as determined by clinical history and confirmed through serology testing, receiving one of the two Moderna (mRNA-1273) or Pfizer-BioNTech (BNT162b2).Our independent validation cohort consisted of a subset (n=16) of study participants from a longitudinal study based in Vancouver, Canada 1. Of the 100 PLWH participants in the Vancouver cohort, 16 were selected based on sample availability and sufficient PBMC count at the time for validation purposes in this study. Ethical approval was granted through the University of British Columbia/ Providence Health Care and Simon Fraser University Research Ethics Boards. Informed consent was obtained from all participants. Vancouver participants had samples collected from baseline (pre-vaccine, V1), one month after SARS CoV-2 mRNA vaccine dose 1 (V2; median 31, range 28-37, Q1-Q3 30-32.5), and one month after vaccine dose 2 (V3; median 30, range 27-32, Q3-Q3 29-30) (Table 1). The time between doses 1 and 2 were median 54, range 49-61, Q1-Q3 51.5-57.5). Participants were recruited to cohorts in New York and Vancouver by self-identification after viewing study recruitment materials in HIV care clinics that outlined the study purpose and major eligibility criteria (persons living with HIV with long-term suppressed viremia who were seeking the SARS-CoV-2 mRNA vaccines) and invited interested persons to contact the study team for more information. Recruited participants broadly reflect the demographics at each recruitment site/country, but otherwise we do not anticipate any overt biases related to recruitment that could impact study results.
Ethics oversight	Ethics approval to conduct this study was obtained from the Institutional Review Boards of Weill Cornell Medicine and the Rockefeller University, or the Research Ethics Board of the University of British Columbia/Providence Health Care and Simon Fraser University. All participants provided written informed consent.

Note that full information on the approval of the study protocol must also be provided in the manuscript.

Clinical data

Policy information about clinical studies

All manuscripts should comply with the ICMJEguidelines for publication of clinical research and a completedCONSORT checklist must be included with all submissions.

Clinical trial registration	N/A - Not an interventional study
Study protocol	Sample collection protocols will be provided upon reasonable request.
Data collection	New York Cohorts: Clinical data were obtained from participants' medical records through EPIC, and compiled between June to August 2021. Clinical data for Vancouver cohort was obtained from the British Columbia (BC) Centre for Excellence in HIV/AIDS Drug Treatment Program Database, which houses clinical records for all PLWH receiving care in BC.
Outcomes	N/A - Not an interventional study

Flow Cytometry

Plots

Confirm that:

- [x] The axis labels state the marker and fluorochrome used (e.g. CD4-FITC).
- [x] The axis scales are clearly visible. Include numbers along axes only for bottom left plot of group (a 'group' is an analysis of identical markers).
- [x] All plots are contour plots with outliers or pseudocolor plots.
- [x] A numerical value for number of cells or percentage (with statistics) is provided.

Methodology

Sample preparation	Peripheral blood mononuclear cells were isolated from leukapheresis material by Ficoll gradient centrifugation.10x106

PBMCs from each study participant collected at visits one, two, and three were thawed and rested for 3 hours at 37 °C 5% CO_2 in R-10 medium (RPMI 1640 supplemented with 10% FBS, L-glutamine, and PenStrep). Additional experimental details are given in the AIM Assay section of "Methods."

Instrument	Attune NxT Flow Cytometer from Thermofisher Scientific
Software	Data were collected with software Attune NxT Software version 3.1, and analyzed with software FLowJo version 10.4.2
Cell population abundance	Shown in Figure 2.
Gating strategy	All samples were initially gated using forward scatter and side scatter to identify events corresponding to lymphocytes. Mononuclear cells were gated out of all events followed by gating live cells as Aqua Fluorescent Dye-, and then subsequent singlet gating. T-cells were gated as CD3+. The AIM+ (CD69+CD137+) CD4+ T-cells or CD8+ T-cells were gated from the CD3+ population, based on an unstimulated (DMSO) control. The entirety of the AIM+ gating for all of the samples studied is shown in Figure 2. The same gates were applied to all samples for any comparison.

☒ Tick this box to confirm that a figure exemplifying the gating strategy is provided in the Supplementary Information.

Software and Code
Data analysis continued:
RNA Seq analysis: Raw reads were quality checked with FastQC v0.11.7 573(http://www.bioinformatics.babraham.ac.uk/projects/fastqc/), and adapters were trimmed using 574Trim Galore v0.6.7 (http://www.bioinformatics.babraham.ac.uk/projects/trim_galore/). Reads 575 were aligned to the human reference genome (GRCh38.p12) using STAR v2.6.0c63 with default 576 parameters. Gene abundances were calculated with featureCounts v1.6.264using composite 577gene models from Gencode release 2865. Principle component analysis was performed using the 578plotPCA function from DESeq2 v1.32.066, after removing patient specific effects with limma 579v3.48.0 removeBatchEffect67. Differentially expressed genes were determined with DESeq2 580v1.32.0 using Wald tests (q < 0.05) with a two-factor model incorporating patient. Gene set 581enrichment analysis was performed using fgsea v1.18.068; genes were ordered between treated 582and untreated cells by the DESeq2 Waldstatistic. Gene sets were retrieved from the Broad 583Institute's MSigDB collections69,70. Only pathways with an adjusted P value < 0.05 were 584considered enriched. The expression heatmap of the leading-edge genes for the MSigDB 585Hallmark TNFA Signaling via NFKB pathway was generated using variance-stabilized data after 586removing patient specific effects, with the values centered and scaled by row.

Antibodies
Validation continued:
Flow cytometry
Biolegend Antibodies:
anti-CD3-Brilliant Violet 785 clone SK7 (cat# 344842)
Each lot of this antibody is quality control tested by immunofluorescent staining with flow cytometric analysis. Additional reported application (for the relevant formats) include: immunohistochemical staining of frozen tissue sections4,5,8, immunofluorescent staining6, and Western blotting3.
References

1. Kan EA, et al. 1983. J. Immunol. 131:536.
2. Wood GS, et al. 1985. Am. J. Pathol. 120:371.
3. Van Dongen JJM, et al. 1988. Blood 71:603. (WB)
4. Haringman JJ, et al. 2005. Arthritis Res. Ther. 7:R862. (IHC)
5. Carbone A, et al. 1999. Blood 93:2319. (IHC)
6. Goval JJ, et al. 2006. J. Histochem. Cytochem. 54:75. (IF)
7. Rutjens E, et al. 2007. J. Immunol. 178:1702.
8. Kap Y, et al. 2009. J. Histochem. Cytochem. 57:1159. (IHC)
9. Yoshino N, et al. 2000. Exp. Anim. (Tokyo) 49:97. (FC)

anti-CD8-BV605 clone RPA-T8 (cat# 301040)
Each lot of this antibody is quality control tested by immunofluorescent staining with flow cytometric analysis. The RPA-T8 antibody does not block the binding of HIT8a antibody to CD8a. Additional reported applications of this antibody (for the relevant formats) include: immunohistochemical staining of paraformaldehyde-fixed frozen sections3 and costimulation of T cell responses4.
References

1. Knapp W, et al. Eds. 1989. Leucocyte Typing IV. Oxford University Press. New York.
2. Schlossman S, et al. Eds. 1995. Leucocyte Typing V. Oxford University Press. New York.
3. Mack CL, et al. 2004. Pediatr. Res. 56:79. (IHC)
4. Magidovich E, et al. 2007. P. Natl. Acad. Sci. USA 104:13022.
5. Thakarl D, et al. 2008.J. immunol. 180:7431. PubMed
6. Kmieciak M, et al. 2009. J. Transl. Med. 7:89. (FC) PubMed
7. Thakral D, et al. 2008. J. Immunol. 180:7431. (FC) PubMed
8. Yoshino N, et al. 2000. Exp. Anim. (Tokyo) 49:97. (FC)
9. Rout N, et al. 2010. PLoS One 5:e9787. (FC)
10. Stoeckius M, et al. 2017. Nat. Methods. 14:865. (PG)

anti-CD137-APC (4-1BB) clone 4B4-1 (cat # 309810)
Each lot of this antibody is quality control tested by immunofluorescent staining with flow cytometric analysis. Additional reported applications (for the relevant formats) include: immunoprecipitation1,4, inhibition of cytokine production2,3, and ELISA.
References

1. Garni-Wagner B, et al. 1996. Cell. Immunol. 169:91. (IP)
2. Salih HR, et al. 2000. J. Immunol. 165:2903. (FA)
3. Kienzle G, et al. 2000. Int. Immunol. 12:73. (FA)
4. Langstein J, et al. 1998. J. Immunol. 160:2488. (IP)

anti-OX40-Brilliant Violet 711 clone Ber-ACT35 (ACT35) (cat # 350030)
Each lot of this antibody is quality control tested by immunofluorescent staining with flow cytometric analysis. Additional reported applications (for the relevant formats) include: Western blotting1, immunoprecipitation1, immunohistochemical staining2,3of paraffin embedded7 and frozen tissue sections, ELISA4, and functional assay5.
References

1. Latza U, et al. 1994. Eur. J. Immunol. 24:677. (WB, IP)
2. Durkop H, et al. 1995. Brit. J. Haematol. 91:927. (IHC)
3. Durkop H, et al. 1997. Brit. J. Haematol. 98:863. (IHC)
4. Willett B, et al. 2007. J. Virol. 81:9665. (ELISA)
5. Li M and Zhang Y. et al. 2005. Cell. Mol. Immunol. 2:467. (FA)
6. Gloviczki ML, et al. 2012. Clin. J. Am. Soc. Nephrol. 8:546. PubMed
7. Domingos PL, et al. 2012. An. Bras. Dermatol. 87:851. (IHC)

anti-CXCR5-AF488 clone J252D4 (cat # 356912)
Each lot of this antibody is quality control tested by immunofluorescent staining with flow cytometric analysis.
References

Wu X, et al. 2016. Sci Rep. 6:36378. PubMed
Punik J, et al. 2021. Cell Report

anti-CD4-AF700 clone A161A1 (cat # 357418)
Each lot of this antibody is quality control tested by immunofluorescent staining with flow cytometric analysis.
References

Mender I, et al. 2020. Cancer Cell. 38(3):400-411.e6. PubMed

anti-human-CD25-APC/Cy7 clone BC96 (cat#302614)
Each lot of this antibody is quality control tested by immunofluorescent staining with flow cytometric analysis. Additional reported applications include: immunocytochemistry3.
References

1. Schlossman S, et al. Eds. 1995. Leucocyte Typing V. Oxford University Press. New York.
2. Kmieciak M, et al. 2009. J. Transl. Med. 7:89. (FC) PubMed
3. Ernst CW, et al. 2007. Clin. Exp. Immunol. 148:271. (ICC) PubMed

Invitrogen antibodies:
Anti-CD69-APC-eFluor 780 clone FN50 (cat# 47-0699-42)
This FN50 antibody has been pre-titrated and tested by flow cytometric analysis of stimulated normal human peripheral blood cells.The FN50 antibody has been reported for use in flow cytometric analysis.
References

Tewari, Ritika et al. "T cell receptor-dependent S-acylation of ZAP-70 controls activation of T cells." The Journal of biological chemistry vol. 296 (2021): 100311. doi:10.1016/j.jbc.2021.100311

Fixable aqua viability dye (cat # L34966)
This kit has been optimized and validated for use with a violet laser flow cytometer.
References:

Ren, Yanqin et al. "Selective BCL-XL Antagonists Eliminate Infected Cells from a Primary-Cell Model of HIV Latency but Not from Ex Vivo Reservoirs." Journal of virology vol. 95,15 (2021): e0242520. doi:10.1128/JVI.02425-20

Beckman coulter antibody:
anti-HIV-1 core antigen p24-RD-1 clone KC57 (cat#6604667)
Validated by the manufacturer based on positive staining of the HIV-infected 8E5 cell line.
References:

Ren, Yanqin et al. "Selective BCL-XL Antagonists Eliminate Infected Cells from a Primary-Cell Model of HIV Latency but Not from Ex Vivo Reservoirs." Journal of virology vol. 95,15 (2021): e0242520. doi:10.1128/JVI.02425-20

ELISPOT
Mabtech antibodies:
anti-IFN-y antibody clone 1-D1K (cat # 3420-2A), anti-Granzyme B antibody clone MT28 (cat # 3486-2A), anti-IFN-y antibody clone 7-B6-1 (cat # 3420-2A), anti-Granzyme B antibody clone MT8610 (cat # 3486-2A)
References:

1. Thomas AS, Jones KL, Gandhi RT, et al. T-cell responses targeting HIV Nef uniquely correlate with infected cell frequencies after long-term antiretroviral therapy. PLoS Pathog. 2017;13(9):e1006629. Published 2017 Sep 20. doi:10.1371/journal.ppat.100662
2. Stevenson, Eva M et al. "HIV-specific T cell responses reflect substantive in vivo interactions with antigen despite long-term therapy." JCI insight vol. 6,3 e142640. 8 Feb. 2021, doi:10.1172/jci.insight.142640
3. Lin, Jonah et al. "Longitudinal Assessment of SARS-CoV-2-Specific T Cell Cytokine-Producing Responses for 1 Year Reveals Persistence of Multicytokine Proliferative Responses, with Greater Immunity Associated with Disease Severity." Journal of virology, vol. 96,13 e0050922. 14 Jun. 2022, doi:10.1128/jvi.00509-22
